# The olfactory secretome varies according to season in female sheep and goat

**DOI:** 10.1186/s12864-019-6194-z

**Published:** 2019-10-30

**Authors:** Paul Cann, Malika Chabi, Aliénor Delsart, Chrystelle Le Danvic, Jean-Michel Saliou, Manon Chasles, Matthieu Keller, Patricia Nagnan-Le Meillour

**Affiliations:** 1 Univ. Lille, CNRS, UMR 8576; USC INRA 1409 - UGSF - Unité de Glycobiologie Structurale et Fonctionnelle, F-59000 Lille, France; 2 ALLICE, 149, rue de Bercy, F-75012 Paris, France; 3 CNRS, INSERM, Univ. Lille, CHU Lille, Institut Pasteur de Lille; Centre d’Infection et d’Immunité de Lille, F-59000 Lille, France; 4 INRA, CNRS, IFCE, Univ. Tours, Physiologie de la Reproduction et des Comportements, F-37380 Nouzilly, France

**Keywords:** Olfactory secretome, Ungulates, Reproduction, Seasonality, Odorant-binding protein, *O*-GlcNAcylation, Phosphorylation, N-glycosylation

## Abstract

**Background:**

Small ungulates (sheep and goat) display a seasonal breeding, characterised by two successive periods, sexual activity (SA) and sexual rest (SR). Odours emitted by a sexually active male can reactivate the ovulatory cycle of anoestrus females. The plasticity of the olfactory system under these hormonal changes has never been explored at the peripheral level of odours reception. As it was shown in pig that the olfactory secretome (proteins secreted in the nasal mucus) could be modified under hormonal control, we monitored its composition in females of both species through several reproductive seasons, thanks to a non-invasive sampling of olfactory mucus. For this purpose, two-dimensional gel electrophoresis (2D-E), western-blot with specific antibodies, MALDI-TOF and high-resolution (nano-LC-MS/MS) mass spectrometry, RACE-PCR and molecular modelling were used.

**Results:**

In both species the olfactory secretome is composed of isoforms of OBP-like proteins, generated by post-translational modifications, as phosphorylation, N-glycosylation and *O*-GlcNAcylation. Important changes were observed in the olfactory secretome between the sexual rest and the sexual activity periods, characterised in ewe by the specific expression of SAL-like proteins and the emergence of OBPs *O-*GlcNAcylation. In goat, the differences between SA and SR did not come from new proteins expression, but from different post-translational modifications, the main difference between the SA and SR secretome being the number of isoforms of each protein. Proteomics data are available via ProteomeXchange with identifier PXD014833.

**Conclusion:**

Despite common behaviour, seasonal breeding, and genetic resources, the two species seem to adapt their olfactory equipment in SA by different modalities: the variation of olfactory secretome in ewe could correspond to a specialization to detect male odours only in SA, whereas in goat the stability of the olfactory secretome could indicate a constant capacity of odours detection suggesting that the hallmark of SA in goat might be the emission of specific odours by the sexually active male. In both species, the olfactory secretome is a phenotype reflecting the physiological status of females, and could be used by breeders to monitor their receptivity to the male effect.

## Background

Small ungulates, such as sheep and goat display distinct annual patterns of reproductive activity that are under photoperiodic control. In females, there is an alternation between periods of ovulatory activity during the short-days of the breeding season (September–January in the northern hemisphere), while the anoestrus period that occurs during the long-days of the spring and summer (February–August in the northern hemisphere) is characterised by ovarian quiescence and anovulation [1]. Interestingly, social factors can override the photoperiodic inhibition of reproductive function during the anoestrus period. Indeed, when sexually active males are joined with anovulatory females at the end of the anoestrus period, a significant proportion of the females will express an immediate reactivation of LH (Luteinizing hormone) pulsatility, which will then lead to ovulation within a few hours [2]. This male-induced ovulation is named the ‘male effect’ or more specifically the “ram effect” in sheep and the “buck effect” in goats. Among the various sensory cues provided by males and that stimulate the reactivation of the gonadotropic axis in females, it has been clearly shown that the male odour is the most efficient one. Indeed, either vocalizations and/or visual cues only induce a small increase in LH-pulsatility and are therefore of low efficiency for the whole reactivation of the gonadotropic axis [3]. By contrast, exposing females to the odour of the male (wool, hair) strongly stimulates LH pulsatility in both sheep and goat [4, 5] and therefore, the effect of the ram or the buck on the LH secretion of females can be mimicked by presentation of their odours [6, 7]. According to the definition of Karlson and Lüscher [8], ram and buck odours could thus be classified as primer pheromones, but several authors reported that goat “pheromones” could induce LH secretion in anoestrus ewes [9, 10]. This apparent lack of specificity is clearly not in favour of a pheromonal action. However, this could more likely suggest common molecules involved in the male effect of these two closely related species. The modulation in pheromone and odours’ perception results on events occurring at every step of the olfactory system, from peripheral to central areas [11]. The central mechanisms involved in the reactivation of the gonadotropic axis have been explored. Olfactory signals have been shown to be processed by the main olfactory system and not by the accessory one, the vomeronasal system [12–14]. The olfactory information is processed then up to the gonadotropin-releasing hormone (GnRH) pulse generator (arcuate nucleus) to reactivate LH pulsatility [14–16]. Meanwhile, the possibility of olfactory plasticity at the peripheral level of olfactory transduction, the first step of odours and pheromones coding, has not been explored so far. Among molecular players, Olfactory-Binding proteins (OBP) bind odorant molecules and deliver them to olfactory receptors [17]. OBP isoforms could also perform the first step of odorant coding by their high binding specificity [18]. Interestingly, it was reported that a family of exocrine gland secreted peptides (ESP) in mouse encodes a VNO-specific ligand repertoire [19], and that expression of these genes is sexually dimorphic and under the control of testosterone. These ESPs display common features with OBPs (secreted by exocrine glands, involved in chemical communication), raising the question whether OBP expression could be sexually dimorphic and dependent on the endocrine status of animals. For example, the sex steroid androstenone of pig (*Sus scrofa*) is abundant in the saliva of sexually active males and induces acceptation of the male during oestrus by females (heat period), and not during di-oestrus. When perceived by pre-pubertal animals, it has appeasing effects and is a signal of submission for piglets [20]. This multiple role in social relationships is due to the endocrine status of the receiver animal, in particular to the level of testosterone that is very low in pre-pubertal individuals of both sexes and in adult females. Recently, we analysed the soluble proteome of nasal mucus in pigs (olfactory secretome) and shown that it is mainly composed of 30 OBP isoforms generated by post-translational modifications (PTM), which are differently expressed in males and females [21]. Moreover, OBP isoforms generated by PTM display very specific binding properties [18], and could differently be expressed according to the endocrinological status of animals during their life. The olfactory secretome could therefore constitute a phenotypic marker of the physiological status of animals, as it is the only part of the olfactory system accessible to non-invasive analyses in living individuals. In this work, we have characterised the olfactory secretome of ewes (*Ovis aries*) and goats (*Capra hircus*), during the season of sexual activity (SA) and during the season of sexual rest (SR), to determine whether each profile is typical and dependent on the seasonal cycle, and if particular OBP isoforms are expressed and secreted in SA period, to enhance the detection ability of male odours by anoestrus females. Indeed, the reactivation of the gonadotropic axis is possibly concomitant with a reactivation of the whole olfactory system and could induce expression of specific OBP isoforms. As an interspecies effect was reported for sheep and goat male odours, we compared the olfactory secretome of females of both species in SR and SA.

## Results

### In silico-predicted OBP sequences of *O. aries* and *C. hircus*

The blast searches for putative ovine and caprine OBPs allowed identification of 16 and 17 sequences, respectively. These sequences were analysed with Signal-P software (DTU Bioinformatics) to remove the signal peptide, the hallmark of secreted proteins, and aligned with Multalin software [22] (Additional file 1: Figure S1). Among these sequences, 18 displayed OBP(*stricto* sensu)-like features (*O. aries:* W5PH68, W5PGV5, W5PZN0, W5PHA2, W5PGN0, W5PHS2, WPPHN1, W5PHM2 and W5PGW3; *C. hircus:* XP_017899539.1, XP_017899538.1, XP_017900101.1, XP_017899208.1, XP_017899536.1, XP_005701296.1, XP_017899515.1, XP_017899207.1, and XP_017899516.1), 8 were close to pig salivary lipocalin (SAL: *O. aries:* W5P8Y1, W5P8W4, W5P4T6 and W5P4W8; *C. hircus:* XP_017908098.1, XP_017908099.1, AHZ46504.1, and XP_017910280.1), and 7 were aligned with Von Ebner’s gland protein (VEG: *O. aries:* W5P559, W5NUS5, and W5NV32; *C. hircus:* XP_005687416.1, XP_017910286.1, XP_017911671.1, and XP_017899201.1). In addition to the typical lipocalin GxW pattern at N-terminal position (14–16 in OBP, 19–21 in SAL, 15–17 in VEG) and the YxxxYxG motif (at position 79–85 in OBP), some common patterns could be observed in some OBP, SAL or VEG-like sequences, but in not all (Additional file 1: Figure S1). In OBP sequences, the most conserved regions are from position 14 to 46 including the GxW motif, and at the C-terminus from residues 151 to 169. In SAL sequences, the GxW hallmark of lipocalins is also included in a well conserved region (12 to 30), whereas in VEG the predicted sequences do not share highly conserved regions. Meanwhile, there is a strong sequence conservation inside each species and between species. It is worth to notice that the number of sequences is much higher in these two ungulate species than in pig and cow (one sequence in each group of OBP, SAL, and VEG). Most of OBP, SAL, and VEG sequences start with a Q at position 1, which can be under either pyroglutamate or glutamate forms in porcine OBP, and possibly modified in ovine and caprine proteins as well. In OBP group, three predicted ovine sequences (W5PH68, W5PGV5, W5PZN0) are more closely related to bovine OBP than to porcine ones, as they have no cysteines at all, and a well-conserved GxW additional motif at position 62–64 instead of the conserved C64 (Additional file 1: Figure S1). These sequences are unable to form disulphide bridges, but could form dimers by domain swapping, as it was reported for bovine OBP [23]. Other sequences were close to porcine OBP (W5PHA2, XP_017899539.1, XP_017899538.1, XP_017900101.1) with 2 cysteines possibly engaged in one disulphide bridge. A sub-group comprised sequences with 2 (W5PGN0, WPPHN1, XP_005701296.1, XP_017899536.1), 3 (XP_017899207.1), or 4 additional cysteines (W5PHS2, XP_017899515.1, XP_017899516.1). The predicted sequence W5PGW3 was not well-aligned with the others, both at the N-terminal end, and along the sequence, but displayed a typical OBP C-terminal end (DDCPA). The SAL group was rather homogenous, with 3–4 conserved cysteines, except sequence W5P4W8 that aligned with other SAL-like from residue D13, but is more divergent in the internal part of the sequence (26% identity with porcine SAL). In the VEG group, two sequences were well-aligned with the porcine VEG (W5NUS5, XP_005687416.1), and W5NV32 was very similar to XP_017910286.1 (96% identity between the two sequences). On the contrary, the caprine sequence XP_017911671.1 seemed truncated at the N-terminal part and did not fit well with other VEG-like proteins.

### Identification of ewe and goat olfactory secretome

We compared the olfactory secretome of 3 females of each species, from samples collected in the same female in SA and SR. All spots obtained after 2D-E of proteins of the nasal mucus were analysed by bottom-up mass spectrometry. Nano-LC-MS/MS or MALDI-TOF MS allowed identifying specific peptides of OBPs among in silico predicted sequences above. It is worth to notice that there is no common peptide between all the OBP sequences in each species and that at least 2 unique peptides were retrieved for each predicted protein identified in analyses below. The mass spectrometry proteomics data have been deposited to the ProteomeXchange Consortium via the PRIDE [24] partner repository with the dataset identifier PXD014833 and 10.6019/PXD014833. Full analyses of raw data are presented in (Additional file 1: Tables S1, S2, S3 for *O. aries* and Tables S4, S5, S6 for *C. hircus*).

#### Olfactory secretome of ewes

**Fig. 1:**
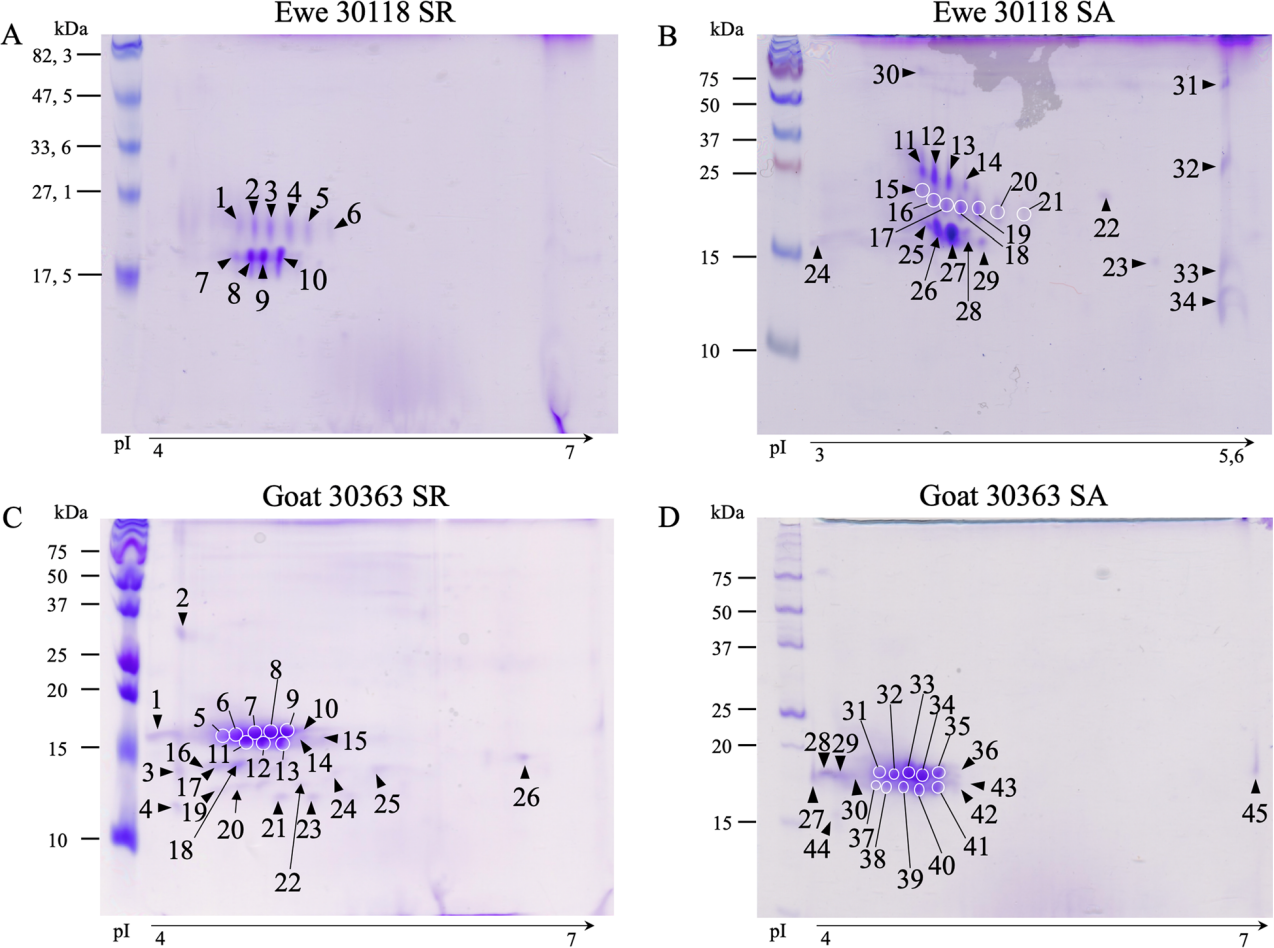
Two-dimensional electrophoresis of soluble proteins extracted from nasal mucus of ewe and goat. Coomassie blue staining. **a** Olfactory secretome of ewe 30,118 in SR, Prestained SDS-PAGE Standards Low Range (Bio-Rad) as molecular weight marker. **b** Olfactory secretome of ewe 30,118 in SA, Precision Plus Protein Standard Dual Color (Bio-Rad). **c** Olfactory secretome of goat 30,363 in SR, Precision Plus Protein Standard All blue (Bio-Rad). **d** Olfactory secretome of goat 30,363 in SA, Precision Plus Protein Standard unstained (Bio-Rad). Protein spots were identified by mass spectrometry (numbering corresponds to Tables 1 and 2; Additional file 1: Tables S1 and S4)

Olfactory secretome profiles obtained by 2D-electrophoresis revealed differences between SA and SR secretomes for the three ewes (Fig. 1a, b & Additional file 1: Figure S2). SR profiles were characterized by two protein strings at apparent molecular masses of 17 (4 spots) and 20 (6 spots) kDa (Fig. 1a). A third string of 5 spots appeared at 25 KDa in SA (Fig. 1b), the 20 kDa string being still composed of 6 spots, whereas the 17 kDa string was composed of 7 spots. Each spot content (numbering in Fig. 1a, b) was identified by nano-LC-MS/MS or MALDI-TOF MS (Table 1). According to their sequence homology with OBP, SAL and VEG already known, the identified proteins were renamed according to the insect OBPs nomenclature: first letter of the genus in capital (e. g. O. for Ovis), three first letters of the species (ari for aries) followed by the type of protein (OBP, e. g. Oari-OBP1). The correspondence between predicted sequence numbers and nomenclature is given in Table 1 for ovine proteins and in Table 2 for caprine ones. In SR the olfactory secretome was mainly composed of specific peptides of two OBP-like proteins that were named Oari-OBP2 (W5PGN0) and Oari-OBP4 (W5PHS2). The 20 kDa string was almost exclusively composed of Oari-OBP2 and the 17 kDa string was composed of a mixture of Oari-OBP2 and Oari-OBP4. Another OBP-like protein was present in only one spot of the 17 kDa string in the ewe 30,094 named Oari-OBP1 (W5PHM2) and a fourth OBP sequence was identified in several spots of the ewe 30,056, Oari-OBP3 (W5PGW3). In ewe 30,056 a mixture of two SAL-like proteins were detected in spots 75 and 81, Oari-SAL1 (W5P8W4) and Oari-SAL2 (W5P8Y1). SA profiles were characterized by a larger diversity, since 7 different protein sequences were identified in all females (Table 1). Oari-OBP2 and Oari-OBP4 displayed the same pattern in 17 and 20 kDa strings than in SR. The additional 25 kDa string mainly contained Oari-SAL1 and Oari-SAL2. The secretomes of ewe 30,094 and 30,056 slightly differed from the ewe 30,118 (Additional file 1: Figure S2), with the two SAL-like expressed in other spots of the 20 kDa string and a mixture of Oari-OBP2 and Oari-VEG1 (W5NUS5) in spots 69 and 70 (ewe 30,094, Additional file 1: Figure S2b). The two other OBP-like proteins (Oari-OBP3 and Oari-OBP1) were found in very few spots in ewes 30,118 and 30,056.

**Table 1:** Proteins identified in spots from SR and SA 2D-E gels of ewes

Protein name *Accession number UniProt KB*	Ewe 30,118 (Fig. 1a, b)		Ewe 30,094 (Additional file 1: Figure S2a, S2b)		Ewe 30,056 (Additional file 1: Figure S2c, S2d)	
	SR^a^	SA^a^	SR^a^	SA^a^	SR¤	SA¤
Protein found in spot n°						
Oari-OBP1 *W5PHM2*	*Not found*	25	45	*Not found*	*Not found*	88 to 90, 92 & 93
Oari-OBP2 *W5PGN0*	1 to 4, 8 to 10	11, 15, 17, 18 & 24 to 29	35 to 38 & 45 to 48	49 to 51, 53 to 56, 58 to 68, 70 & 71	72, 73 & 76 to 83	84 to 86, 89 & 93 to 103
Oari-OBP3 *W5PGW3*	*Not found*	18 to 21	*Not found*	60	72, 78 & 81 to 83	87, 88, 95, 98, 99 & 101
Oari-OBP4 *W5PHS2*	1, 8 & 9	11, 12, 15, 17, 18 & 24 to 28	35, 45 & 46	51, 55, 56, 58, 61 to 68 & 71	72 to 83	93, 98 to 100
Oari-SAL1/2 *W5P8W4 / W5P8Y1*	*Not found*	11 to 15	*Not found*	49 to 53, 55, 56, 58 & 60	75 & 81	84 to 89, 91 & 99
Oari-VEG1 *W5NUS5*	*Not found*	*Not found*	*Not found*	69 & 70	*Not found*	*Not found*

^a^proteins identified by Nano-LC-MS/MS ¤ Proteins identified by MALDI-TOF MS. Full data can be found in of Additional file 1: Tables S1, S2 and S3

**Table 2:** Proteins identified in spots from SR and SA 2D-E gels of goats

Protein name *Accession number GenPept*	Goat 30,363 (Fig. 1c, d)		Goat 30,422 (Additional file 1: Figure S3a, S3b)		Goat 30,432 (Additional file 1: Figure S3c, S3d)	
	SR^a^	SA¤	SR¤	SA¤	SR^a^	SA^a^
Protein found in spot n°						
Chir-OBP1 *XP_017899536.1*	*Not found*	*Not found*	*Not found*	*Not found*	85	124, 129 & 144
Chir-OBP2 *XP_017899208.1*	1, 2, 5 to 15, 17, 20, 22, 24 & 25	27 to 29, 31 to 43 & 45	46 to 64, 66 to 70 & 73	74 to 82	83 to 105	108, 109, 121, 122, 124, 126 to 134, 136, 137, 139, 140 & 142 to 145
Chir-OBP3 *XP_005701296.1*	*Not found*	30 to 32	47 to 49, 51 to 54, 57, 59, 62, 66 & 67	75, 76 & 80	*Not found*	127
Chir-OBP4 *XP_017899515.1*	1 to 3, 5 to 15 & 17 to 19	31 to 35, 38, 40, 41 & 45	46 to 48, 51 to 63, 65 & 73	76 & 78	83 to 95 & 97 to 104	108 to 114, 117 to 119, 122 to 124, 127 to 134, 136 to 138, 140, 142, 144 & 145
Chir-OBP5 *XP_017899538.1*	*Not found*	28, 30 & 32	46, 47, 50, 52 to 56, 59, 60, 64, 68, 70 & 71	76, 78 & 79	86	121 & 122
Chir-OBP6 *XP_017900101.1*	1	28 to 31, 36, 37 & 45	47, 49 & 72	74 & 76 to 78	86	*Not found*
Chir-SAL1/2 *XP_017908099/8.1*	2	31, 32, 35, 41, 43 & 44	46 to 48, 50 to 57, 59 to 67, 69, 71 & 72	74, 77, 78 & 80 to 82	87 & 90	*Not found*
Chir-VEG1 *XP_005687416.1*	10	30 & 34	54, 55 & 63	*Not found*	*Not found*	*Not found*
Chir-VEG2 *XP_017911671.1*	11	32, 35 & 39 to 43	46 to 67, 69, 71 & 73	78 & 80 to 82	97 to 100	134
Chir-VEG3 *XP_017910286.1*	*Not found*	32 & 38	*Not found*	74 & 77	*Not found*	*Not found*

^a^proteins identified by nano-LC-MS/MS ¤ Proteins identified by MALDI-TOF MS. Full data can be found in Additional file 1: Tables S4, S5 and S6

#### Olfactory secretome of goats

The olfactory secretome profiles of goats in SR (Fig. 1c**,** Additional file 1: Figure S3a, S3c) revealed a major protein string of zip shape at an apparent molecular mass of 17 kDa and a smaller string at 15 kDa. The SA profiles were similar to the SR ones for the three goats (Fig. 1d**,** Additional file 1: Figure S3b, S3d), even if the profiles of goat 30,363 (Fig. 1c, d) differed slightly from those of the two other animals (Additional file 1: Figure S3), by the lack of the 15 kDa string. In SR samples, the strings at 17 kDa and at 15 kDa were mainly composed (numbering in Fig. 1c, d) of Chir-OBP2 (XP_017899208.1) and Chir-OBP4 (XP_017899515.1) specific peptides for the three goats (Table 2). But contrary to ewes SR profiles, several other proteins could be identified: Chir-VEG2 (XP_017911671.1) is present in few spots in the three females, whereas Chir-OBP3 (XP_005701296.2), Chir-OBP5 (XP_017899538.1) and Chir-VEG1 (XP_005687416.1) are present only in goat 30,422. Chir-SAL1 (XP_01708099.1) and Chir-SAL2 (XP_01708098.1) were detected in mixture in some spots of this string in this female (Table 2). The spot in the basic part of the gel in goat 30,422 profile (spot n°73, Additional file 1: Figure S3a) contained specific peptides of Chir-OBP2, Chir-OBP4 and Chir-VEG2 isoforms. Chir-OBP2 and Chir-OBP4 were the major components of the 17 kDa string in SA, with some isoforms of Chir-OBP3 and Chir-VEG2 for the three females. For the goat 40,322 the Chir-OBP4 was not as present as in the other females. Specific peptides of Chir-OBP5, Chir-OBP6, Chir-SAL1, Chir-SAL2 were identified in this string, except in goat 30,432. In addition, peptides of two new proteins were identified in the 17 kDa string: Chir-OBP1 (XP_017899536.1) in goat 30,432, Chir-OBP6 (XP_017900101.1) and Chir-VEG3 (XP_017910286.1) in goats 30,363 and 30,422. As well as the majority of spots in these profiles, the 15 kDa spots in SA (spot 139 to 143 of goat 30,432) contained specific peptides of Chir-OBP2 and Chir-OBP4, and basic spots of SA profiles (spots 144 & 145 of goat 30,432), except for spot n°144 of the goat 30,432 that contained peptides of Chir-OBP1 (Table 2**,** Additional file 1: Figure S3d) Spots of the 25 kDa string, specific to goat 30,432, contained mainly Chir-OBP2 and Chir-OBP4 in both SR and SA, plus some peptides of Chir-SAL1 in SR, and Chir-OBP5 in SA.

#### Comparison between the 2 species

The olfactory secretome profiles of ewe and goat differed in the number of detected spots and in the number of protein strings, but share a common main string at apparent molecular mass of 17 kDa. The interindividual variability in goats participated to this difference between the two species. The composition of theses profiles was also different, as more proteins are present in goat than in ewe in both SR and SA. In goats, six OBP-like proteins were identified, instead of four in ewes, 3 VEG-like in goat and only 1 in ewe, but two SAL-like proteins were identified in both species. The olfactory secretome profile of ewe is remarkably similar to the pig’s one [21], where SAL-like proteins are dispatched in a protein string with a decreasing molecular weight gradient from acid to basic spots, and with VEG proteins in the basic part of the main spots (17 kDa string in ewe, Additional file 1: Figure S2b). Even if the distribution of these protein families is not as clear in goat as in ewe, the olfactory secretomes are mainly composed of isoforms of two OBP-like sequences: Oari-OBP2 and Oari-OBP4 in ewe, and Chir-OBP2 and Chir-OBP4 in goat. These proteins were identified in almost all spots whenever the season in the 2 species, and remarkably close to each other in the previous alignment (Additional file 1: Figure S1). To confirm this phylogenetic proximity between the two OBP2 and the two OBP4, the nucleotide sequences were amplified from ewe and goat olfactory epithelium.

### Amplification and molecular cloning of nucleotide sequences coding for OBP2 and OBP4

#### cDNA analysis of ovine and caprine main sequences

**Fig. 2:**
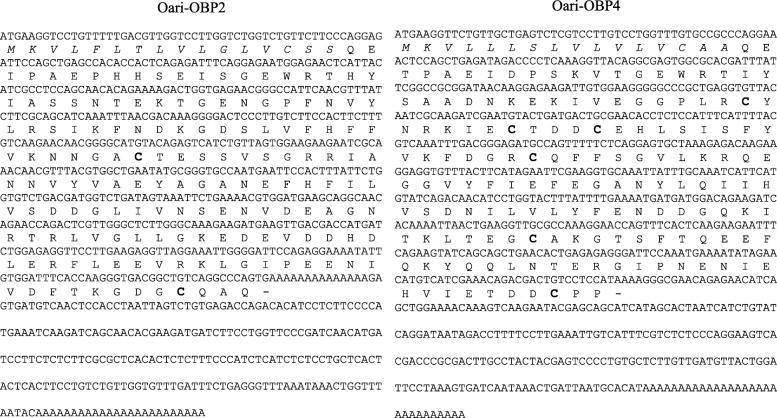
Nucleotide and deduced amino acid sequences of Oari-OBP2 and Oari-OBP4. Signal peptide is in italics and cysteines in bold

**Fig. 3:**
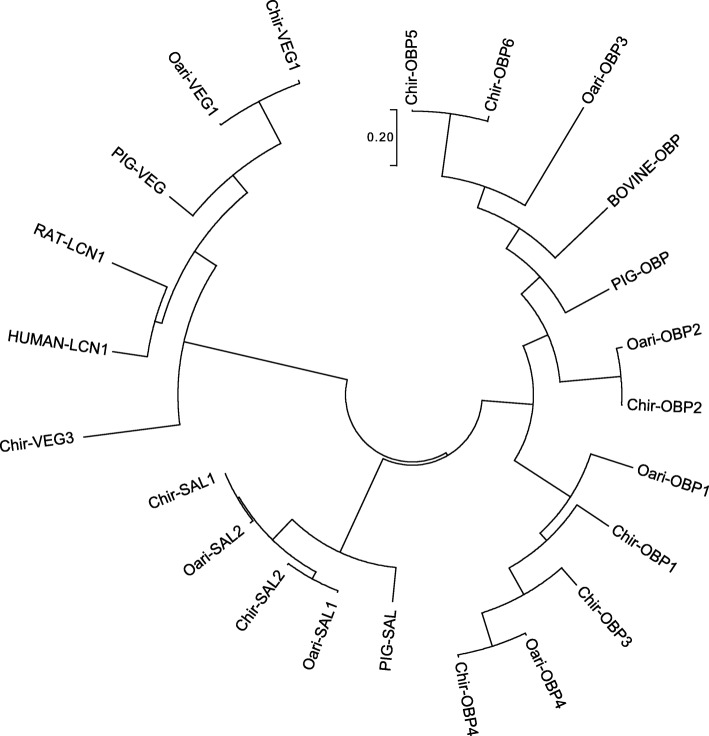
Phylogenetic analysis by Maximum Likelihood method of OBP-like nucleotide sequences. GenBank accession number of cloned sequences: *Oari-OBP2*: MK908984; *Oari-OBP4*: MK908985; *Chir-OBP2*: MK908982; *Chir-OBP4*: MK908983. Ensembl Ovine transcript ID: *Oari-OBP1*: ENSOART00000010082.1; *Oari-OBP3*: ENSOART00000009820.1; *Oari-SAL1*: ENSOART00000006976.1; *Oari-SAL2*: ENSOART00000006993.1 and W5NUS5: ENSOART00000001966.1. Caprine NCBI Reference Sequence: *Chir-OBP1*: XM_018044047.1; *Chir-OBP3*: XM_005701239.1; *Chir-OBP5*: XM_018044049.1; *Chir-OBP6*: XM_018044612.1; *Chir-SAL1*: XM_018052610.1; *Chir-SAL2*: XM_018052609.1; *Chir-VEG1*: XM_005687359.2; *Chir-VEG2*: XM_018056182.1 and *Chir-VEG3*: XM_018054797.1. Other NCBI Reference Sequence: *PIG-OBP*: NM_213796.1; *BOVINE-OBP*: XM_002700469.6; *PIG-SAL*: NM_213814.2; *PIG-VEG*: NM_213856.2; *RAT_LCN1*: NM_022945.1 and *HUMAN_LCN1*: NM_001252617

The cDNA encoding the 2 most abundant OBPs, OBP2 and OBP4, identified in ewe and goat secretome were amplified by RACE-PCR. Indeed, the predicted sequences available from databases are unverified, often automatically deduced from high-throughput sequencing data (e. g. contigs assembly) and should be amplified from tissues to ascertain the sequence. The full-length sequences (GenBank accession numbers: MK908982, MK908983, MK908984 and MK908985), when translated, showed high identity with the predicted Oari-OBP2 and Oari-OBP4 (Fig. 2), Chir-OBP2 and Chir-OBP4 (Additional file 1: Figure S4). Oari-OBP2 was 98% identical to the predicted sequence, the main differences were located in the middle of the sequence with two frameshifts modifying the sequence (deleting C51 and C56 in the predicted), and at the 3′ end modifying the C-terminal sequence (GDCSLA - predicted and GCQAQ - cloned). For the three other sequences the identity between predicted and cloned sequences were higher (Oari-OBP4: 99%, Chir-OBP2: 99.4% and Chir-OBP4: 100%). These four sequences were added to the predicted olfactory-binding proteins and reference sequences to perform a Blast search in order to build a phylogenetic tree (Fig. 3). As expected from the predicted sequences, ovine and caprine sequences segregated into the three OBP, VEG and SAL groups. The tree revealed the high proximity between sheep and goat sequences inside protein families, e. g. between Chir-VEG1 and Oari-VEG1, Oari-SAL1 & SAL2 and Chir-SAL1 & SAL2.

#### Comparison of sheep and goat sequences

**Fig. 4:**
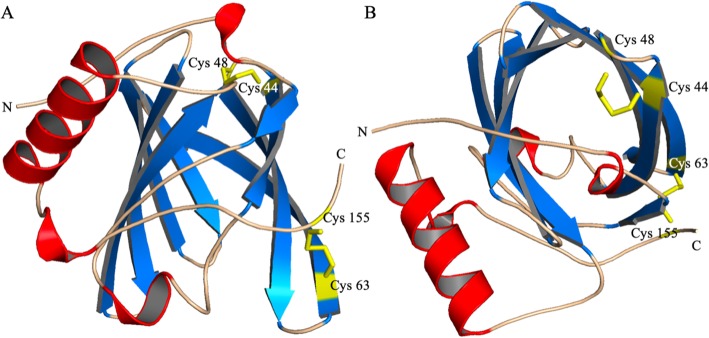
Ribbon representation of Oari-OBP4 3D structure predicted by homology modelling. The β-strands are coloured in blue, α-helix in red, loops in wheat and cysteines involved in disulphide bridges in yellow. **a** side views. **b** view from above

Chir-OBP2 and Oari-OBP2 shared 97.72% identity of nucleotide sequence (98.3% of homology and 96% of identity between protein sequences), whilst Chir-OBP4 and Oari-OBP4 shared 98.1% identity at the nucleotide level (96.5% of homology and 95.3% of identity at the protein sequence level). Such identities are unusual for OBPs of different species, generally 20% of similarities are observed between species [17]. This suggests that OBP2 and OBP4 could have a common role in odours reception of the two species. The two OBP2 sequences are close to porcine OBP in the phylogenetic tree (Fig. 3) and share 58% of similarity, and could display common binding properties for steroids and fatty acids [18]. This is supported by the molecular modelling experiments performed to predict the 3D-structure of these sequences. The predicted model by I-TASSER software [25] showed that the four sequences have a 3D-structure typical of lipocalins, with eight β-strands forming a β-barrel in addition to an α-helix, and a disulphide bridge (Cys63-Cys155) conserved in all Mammalian OBPs except in the bovine one [17]. If the two OBP2 have a 3D-structure very similar to porcine OBP [26], a second disulphide bridge is formed in the OBP4 between Cys44 and Cys48 (Fig. 4). This bridge is only found in rodents OBP, more specifically in Cricetidae, in proteins expressed in fluids involved in chemical communication, such as hamster aphrodisin [27] and bank vole glareosin [28]. In aphrodisin, the disulphide bridge forms a loop where an asparagine, not conserved in OBP4, is N-glycosylated [29]. Hence, the two OBP4 seem rather specific to the small ungulates, and could have a role in the binding of specific odours of these species. It could be the same for the Chir-SAL1 & SAL2 and Oari-SAL1 & SAL2 which are very close in the tree, and known to be Pheromone-Binding Proteins [30]*.* Their specific expression in SA in ewe could reflect an adaptation of female secretome to odours emitted by a sexually active ram. In goat, SAL are expressed in SR and not systematically present in SA, in this case the modification of the secretome could result on the increasing number of isoforms of OBP sequences in SA. In particular, new isoforms could be generated by post-translational modifications (PTM), as it was evidenced in pig [21].

### Sheep and goat olfactory-binding proteins are modified by post-translational modifications

#### N-glycosylation

**Fig. 5:**
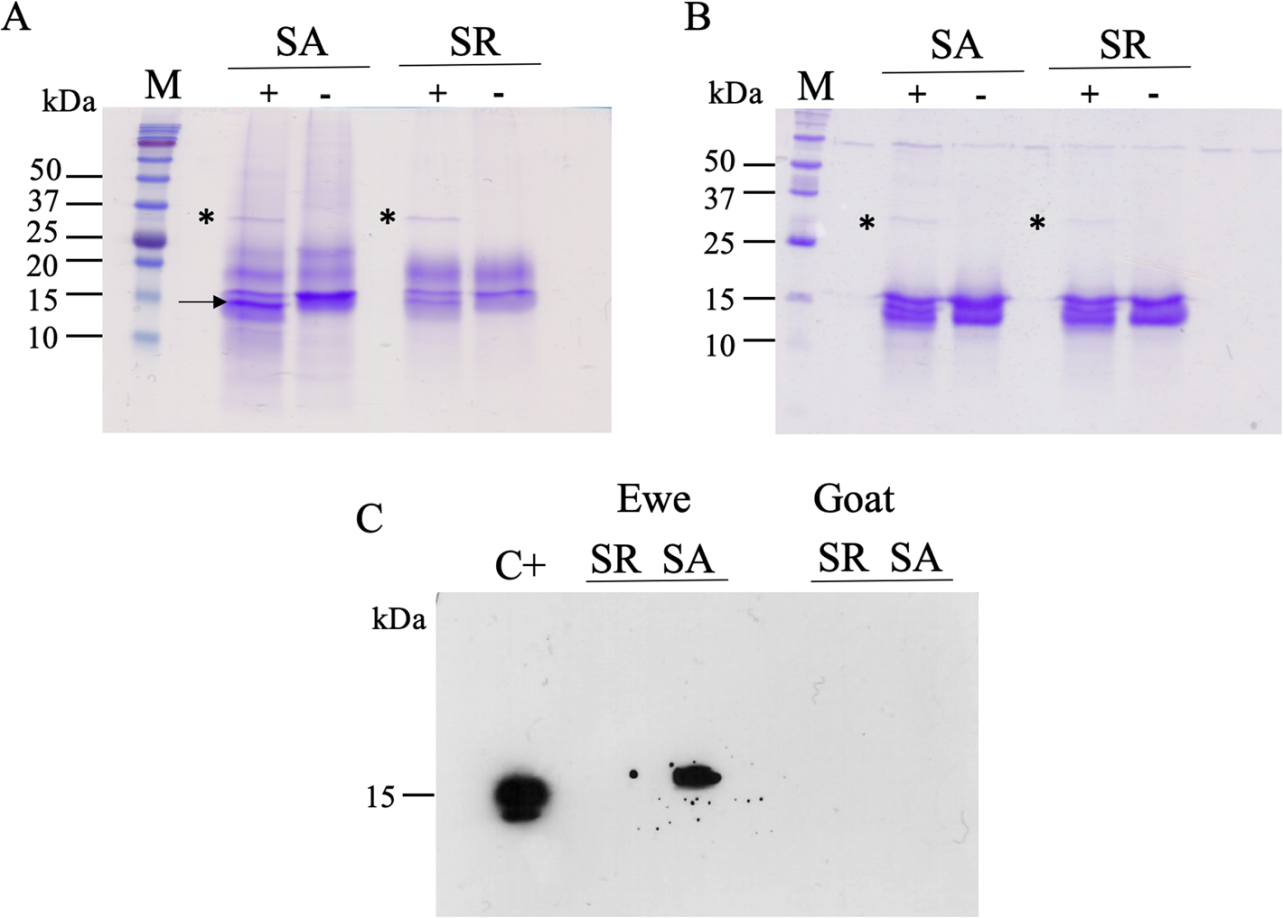
Detection of N-Glycosylation on proteins of the olfactory secretome of ewe and goat. Total soluble proteins extract (15 μg each) from nasal mucus of ewe 30,094 in SA and SR (**a**) and goat 30,422 in SA and SR (**b**), with (+) and without (−) PNGase F digestion (* indicates the enzyme), Coomassie blue staining. **c** ECL detection of internal mannose by lectin-blot with ConA-perox lectin (ewe 30,118 and goat 30,063), C+: positive control is 500 ng of RNase B)

Samples were treated with PNGase F, an enzyme that specifically removes N-glycan chains from proteins (Fig. 5). A clear shift in molecular mass was observed only in treated sample of ewe in SA, characterized by the emergence of an additional band compared to the non-treated sample (arrow, Fig. 5a). This decrease in molecular mass suggests that one or several glycan chains were removed by PNGase F from the band migrating at 25 kDa (spots 11–14 in Fig. 1b) and/or from the band migrating at 20 kDa (spots 15–21, Fig. 1b) in SA, from proteins of the 17 kDa string in SR (spots 7–10, Fig. 1a). To confirm that one or more proteins bear glycan-chain, a lectin-blot with Con A lectin was performed on the non-treated samples (Fig. 5c). For the goat samples and the sample of ewe secretome in SR, no signal was obtained. The reactivity to Con A lectin indicated the presence of poly α-mannoses chain(s) on ewe proteins. The strong signal in SA could be due to Oari-SAL1 and Oari-SAL2 that are not expressed in SR (Table 1). Indeed, pig SAL, which is phylogenetically close to Oari-SALs (Fig. 3) is N-glycosylated [31]. Four sites of N-glycosylation were indeed predicted by the NetNGlyc software (http://www.cbs.dtu.dk/services/NetNGlyc/) for Oari- and Chir-SAL. These results suggest the existence of two groups of SAL-like proteins in ewe, one group of isoforms in the 25 kDa string in SA, N-glycosylated, and one group of isoforms in the 20 kDa string (Table 2), which are not N-glycosylated. As one site of N-glycosylation was predicted by NetNGlyc for Oari-OBP2, its isoforms in the 20 kDa string could be N-glycosylated as well.

#### Phosphorylation

**Fig. 6:**
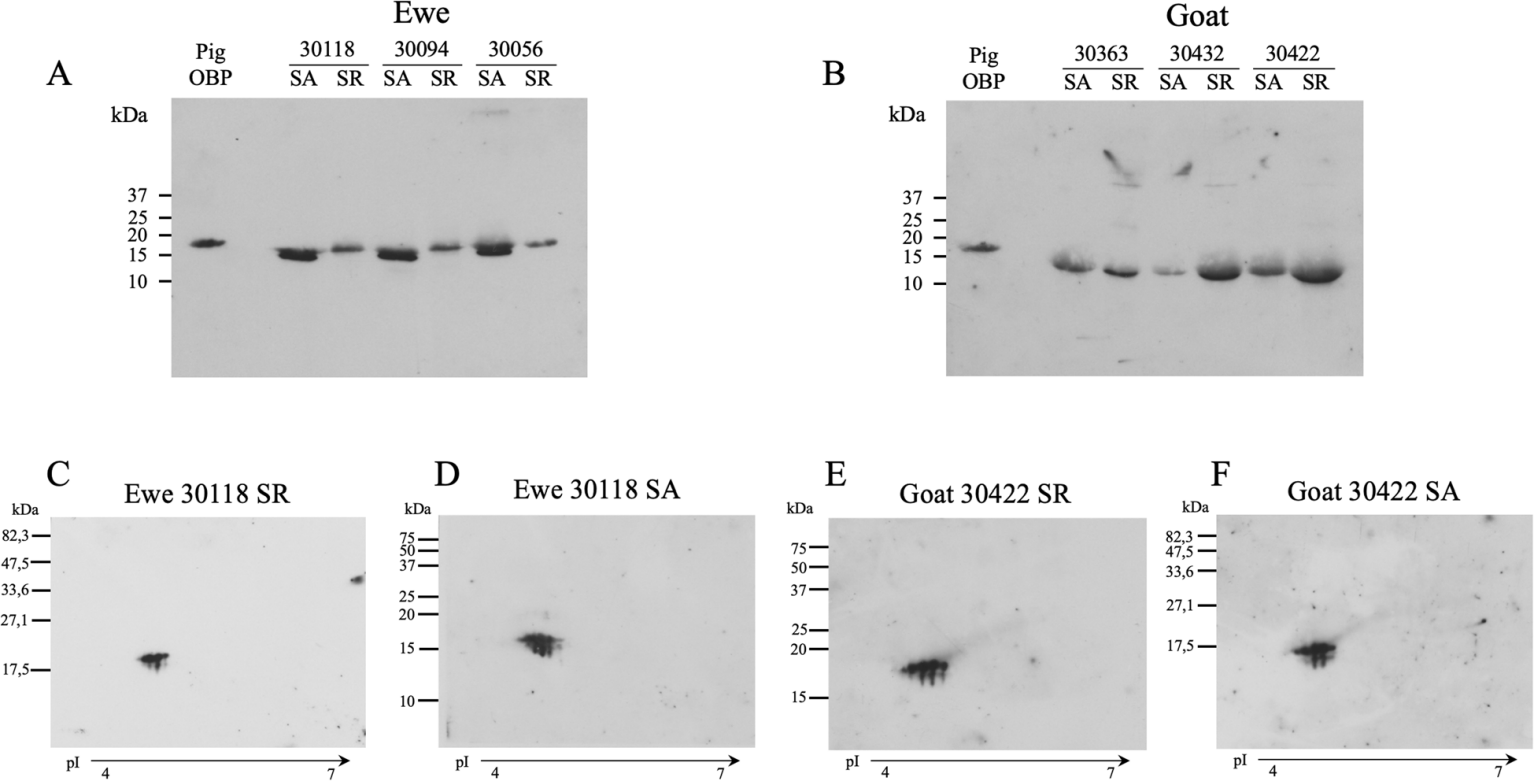
Detection of phosphoserine(s) on proteins of the olfactory secretome of ewe and goat. ECL detection of proteins phosphorylated on serine by western-blot with anti-phosphoserine Q5 Antibody (Qiagen). **a** SDS-PAGE of ewes 30,118, 30,094, 30,056 samples in SA and SR. **b** SDS-PAGE of goats 30,363, 30,422, 30,432 samples in SA and SR. **c** 2D-E of ewe 30,118 sample in SR. **d** 2D-E of ewe 30,118 sample in SA. **e** 2D-E of goat 30,422 sample in SR. **f** 2D-E of goat 30,422 sample in SA. Pig OBP (15 μg) was used as positive control [32]

The OBP phosphorylation was investigated on ewe and goat proteins of olfactory secretome, as previous work has shown that porcine olfactory-binding proteins are phosphorylated [18, 32]. Anti-phosphoserine antibody labelled all samples from ewe and goat, whenever the season (Fig. 6). In ewes, two bands were labelled in SA and only one in SR (Fig. 6a). In goat, labelling of SR samples seemed stronger than in the SA samples (Fig. 6b). The western-blot after 2D-E showed that, in ewe in SR, 4 spots of the main string at c. a. 17 kDa were labelled (Fig. 6c), whereas 5 spots of the same protein string are labelled in SA (Fig. 6d). In goat, the same number of spots were labelled in SR and SA samples (9 spots of the 17 kDa string, Fig. 6e, f). Western-blot and Coomassie blue stained gels of 2D-E were merged, which allowed identification of the labelled spots. The map of labelled spots can be found in Additional file 1: Figure S5 (a-d). The same samples were digested with alkaline phosphatase to assess the specificity of Q5 antibodies (Additional file 1: Figure S6a). Indeed, dephosphorylated samples were not labelled by this antibody after western-blot. Comparing the proteins identified in these spots and the antibody labelling, we propose that OBP2 and OBP4 could carry this modification in both species. The physiological status of the female could have an impact on the number of phosphorylated isoforms expressed in the secretome, leading to more phosphorylation in SA in ewe and more phosphorylation in SR in goat. Conversely, antibody Q7, raised against phosphothreonine labelled all samples (Additional file 1: Figure S7).

To confirm and if possible, localize the phosphorylation sites in ewe and goat OBPs, nano-LC-MS/MS data were analysed with Scaffold software. Ion scores, identity scores and retention times were compared between phosphorylated and naked spectra for each phosphorylated peptide (spectra of peptide 18–40 of Oari-OBP2 are compared in Additional file 1: Figure S8). Despite the immunodetection of potential phosphorylated serine in both species (Fig. 6) no corresponding sites were identified by mass spectrometry, contrary to phosphothreonine sites identified in accordance with the labelling in western-blot (Additional file 1: Figure S7). In ewe, phosphorylation sites were identified on Oari-OBP2, Thr18, Thr29 and Th65, only in SA. Thr18 site was confirmed in spot 67 of ewe 30,094 (Additional file 1: Figure S2b) by 2 spectra, Thr29 site by one spectrum in spot 27 of ewe 30,118 (Fig. 1b), and Thr65 site in ewe 30,094 (spot 68, Fig. 1b) and 30,118 (spot 32, Additional file 1: Figure S2b) by 2 spectra for each spot. Except for spot 32 of ewe 30,118, all spots were located in the 17 kDa string. In goat, 3 sites were identified on Chir-OBP2, Thr29, Tyr38 and Thr64, with some interindividual differences. In goat 30,432 in SA (Additional file 1: Figure S3d), Tyr38 site was identified by 2 spectra on spots 138 and 140. In SR, Thr29 site was identified by 6 spectra in goat 30,363 (spots 6 to 9, Fig. 1c) and by 3 spectra in goat 30,432 (in spots 100, 101, and 105, Additional file 1: Figure S3c). Tyr38 site was phosphorylated on peptides found in many spots of goat 30,363 (SR, Fig. 1c) by eleven spectra in spots 5 to 9, and 11. In goat 30,432 (SR, Additional file 1: Figure S3c), Tyr38 site was identified by 9 spectra of two different peptides in spots 99, 100, 105, and 106. Thr64 was identified in spot 8 of goat 30,363 in SR by three spectra. The spots where Thr29 site was identified belonged, in the two goats in SR, to the 17 and 15 kDa strings. In SA of goat 30,432, Tyr38 was phosphorylated only in spots at 15 kDa or lower. In SR, Tyr38 site was found in spots of the 17 kDa string in goat 30,363, and in goat 30,432 except for spot 106 (Additional file 1: Figure S3c) which is located in a more basic part of the 2D-E gel. The fact that no site of phosphoserine was identified by mass spectrometry, despite the strong labelling of specific antibody is surprising regardless the literature, since phosphorylation of serine is described as the most abundant phosphorylation in Mammals (ratio Ser/Thr/Tyr of 1800/200/1) [33]. This could come from the lability of this modification submitted to the high energy of HCD ionization, and the possibility of serine modification cannot be excluded. As porcine OBP is phosphorylated [32], the identity between the primary sequences of porcine OBP, Oari-OBP2 and Chir-OBP2 led us to compare the phosphorylation pattern of these sequences. Recently, phosphorylation sites on porcine OBP were identified by CID-nano-LC-MS/MS [18]. Several sites are common between the 3 species (Ser13, Ser23, Ser24, Ser41, and Ser67) and are predicted as phosphorylated by the NetPhos server (http://www.cbs.dtu.dk/services/NetPhos/). Thr112, which is phosphorylated in pig, was not detected as a phosphorylation site in sheep and goat sequences (Additional file 1: Figure S1).

#### O*-GlcNAcylation*

**Fig. 7:**
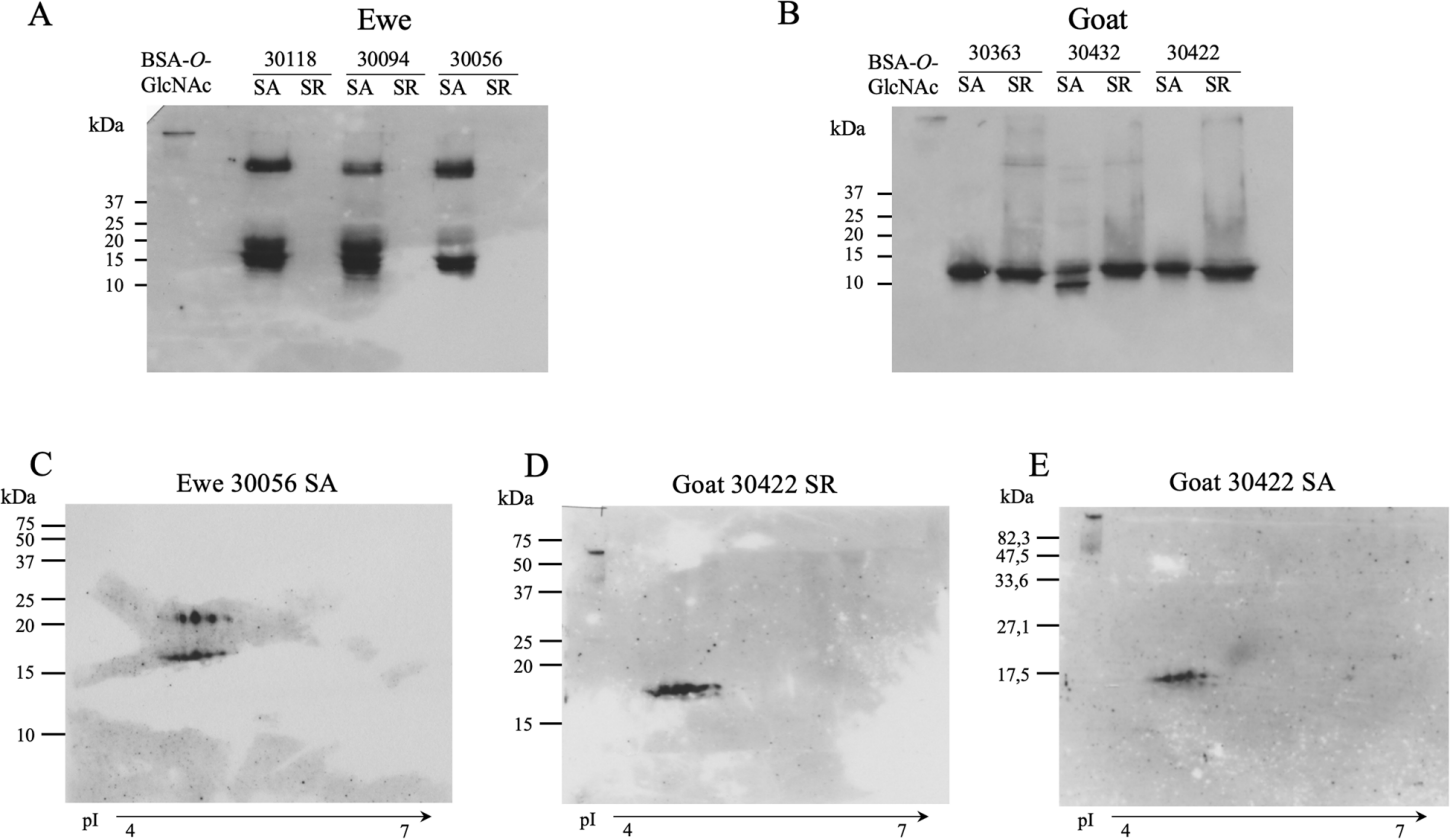
Detection of *O-*GlcNAcylation of proteins of the olfactory secretome of ewe and goat. ECL detection of *O-*GlcNAcylated proteins by western-blot with CTD110.6 Antibody. **a** SDS-PAGE of ewes 30,118, 30,094, 30,056 samples in SA and SR. **b** SDS-PAGE of goats 30,363, 30,422, 30,432 samples in SA and SR. **c** 2D-E of ewe 30,118 sample in SA. **d** 2D-E of goat 30,422 sample in SR. **e** 2D-E of goat 30,422 sample in SA. Positive control: 5 ng of GlcNAc-modified BSA (Fisher Scientific)

A major difference between the two species appeared with the detection of *O*-GlcNAcylation on OBP. In goat (Fig. 7b), the samples of the three females in SA and SR were labelled by CTD110.6 antibody, suggesting an equal level of *O*-GlcNAcylation. For the three ewes, only the SA samples were labelled, no *O*-GlcNAcylation was detected in SR (Fig. 7a). The labelling at a higher molecular mass could correspond to oligomers frequently observed for OBPs [21], which could be more reactive to antibodies than monomers, as a result of their tendency to form oligomers in solution. Western-blot performed after 2D-E brought more information on the number of *O*-GlcNAcylated spots: in ewe (in SA) four spots of the 25 kDa string were labelled and five spots of the 17 kDa string (Fig. 7c). In the SR sample of goat, 8 spots of the 17 kDa string were labelled (Fig. 7d), and 6 spots of this string in SA (Fig. 7e). The map of labelled spots can be found in Additional file 1: Figure S5 (e-g). The competition assay between CTD110.6 antibody and free GlcNAc assessed the specificity of the antibody (Additional file 1: Figure S6b) and *O*-GlcNAcylation of OBPs in ewe and goat olfactory secretome. The HCD-nano-LC-MS/MS is not a suitable method to identify *O*-GlcNAcylation sites, as GlcNAc moieties are removed during ionization. However, spots were specifically labelled by CTD110.6 antibody (Fig. 6, Additional file 1: Figure S5f, S5g) suggesting in goat that Chir-OBP2 and Chir-OBP4 could be *O*-GlcNAcylated as their isoforms were identified in all labelled spots. In ewe, isoforms of Oari-OBP2 and Oari-OBP4 of the 17 kDa string could carry this modification as well, in addition to Oari-SAL1 and SAL2 which mainly composed the 25 kDa string (Fig. 6e). The sequence similarity between Oari-OBP2, Chir-OBP2 and porcine OBP reinforces this hypothesis, as porcine OBP is *O*-GlcNAcylated [21] on Ser13 and Thr18 [18] that are conserved in sheep and goat OBP2 sequences. Even if Oari-OBP4 and Chir-OBP4 are phylogenetically more distant to the porcine OBP (Fig. 3), the number of their isoforms in the labelled spots does not totally exclude the *O-*GlcNAcylation of these proteins. The potential *O*-GlcNAcylation of SAL-like proteins (both in sheep and goat) is supported by the similarity between these sequences and the porcine SAL (64% of similarity), which is *O*-GlcNAcylated [21]. VEG-like sequences found in the spots labelled by CTD110.6 could also be modified by GlcNAc [34], but it has to be confirmed by ETD-nano-LC-MS/MS, a technique more suitable to localize *O*-GlcNAcylation sites [35].

## Discussion

### The olfactory secretome of ewe and goat is composed of numerous isoforms of new OBP sequences

The sampling of nasal mucosa with a new non-invasive protocol allowed deciphering the olfactory secretome of ewe and goat. It is composed of OBP sequences described for the first time in these species, together with their post-translational modifications. The sequences are very similar for lipocalins of this family, reflecting the phylogenetic proximity between sheep and goat, and a common genetic background tuned to olfaction. The comparison of secretomes between the two species and between the two endocrinological status of SR and SA bring information on the molecular modalities involved in the adaptation of the sensory equipment to physiological changes during animal life. The olfactory secretome of ewe is characterised by an important change in SA, consisting in the expression of SAL-like, probably N-glycosylated, an increase in serine phosphorylation, and the emergence of *O*-GlcNAcylation, these three modifications generating new isoforms, specific to SA. In goat, the composition of olfactory secretome is apparently less contrasted between the two seasons than in ewe. Meanwhile, a fine analysis reveals new protein sequences expressed, and a decrease in serine phosphorylation. Furthermore, some variations in the post-translational modifications could not be evidenced by this work, as the pattern of PTM can vary by the modification of different sites along the primary sequence, without any impact on the global signal observed in western-blot. This could be the case for the *O*-GlcNAcylation signal of goat samples that is identical in both seasons, but could result on the modification of different sites in the OBP sequences. Hence, the variation in composition of olfactory secretome occurs in both species in sexual activity period, by the expression of new OBP sequences and the modulation of the PTM, generating a set of isoforms specific to the SA.

### Post-translational modifications of olfactory binding proteins: a new metabolic pathway in mammalian olfaction?

N-glycosylation is a typical post-translational modification of proteins processed in the secretory pathway [36]. Several lipocalins involved in chemical communication are N-glycosylated, e. g. the female hamster aphrodisin [37], and the porcine SAL [31]. Conversely, phosphorylation and *O*-GlcNAcylation of secreted proteins are less conventional, these PTM were previously described as modifying nuclear and cytoplasmic proteins. Phosphorylation of secreted proteins is now well-documented [38], and has been reported as a modification of OBP, first on porcine OBP [18, 21, 32], and recently on panda OBP (*Ailuropoda melanoleuca*) [39]. The underlying mechanisms remain unknown, although a member of the casein kinase family, FAM20C, could be a good candidate to phosphorylate these proteins, even if the consensus motif S-x-E/pS is not found in all predicted phosphorylation sites of Oari-OBP2 and Chir-OBP2. Indeed, FAM20C phosphorylates more than 100 extracellular proteins [38] in Golgi apparatus of the secretory pathway [40]. Other candidate enzymes could be Ecto-PK1 and Ecto-PK2, which phosphorylate both extracellular and membrane proteins in the extracellular compartment [41]. OBPs of sheep and goat could be phosphorylated either in the secretion pathway by a Golgi enzyme, or after their secretion by an extracellular kinase. OBP *O*-GlcNAcylation was only reported yet for porcine species [18, 21], but its evidence in ovine and caprine species extends the possibility that this modification could be found in all Mammalian species. In ewe, the lack of labelling in SR compared to SA questions about a possible hormonal control of *O*-GlcNAcylation. In addition, the equal labelling of goat isoforms is apparent and could not reflect the *O*-GlcNAcylation of different isoforms in the same spots. It was proposed that porcine OBP *O*-GlcNAcylation [21] could be performed by a ER-resident enzyme, primarily identified in Drosophila [42], the EOGT glycosyltransferase (EGF-domain *O*-linked N-acetylglucosaminyl transferase). The encoding gene is present in sheep and goat genome at accession numbers W5PKW4 and XP_017893891.1, respectively. The porcine, ovine and caprine EOGT protein sequences share 95% of homology suggesting a common role in OBP *O-*GlcNAcylation.

### Is the olfactory secretome a phenotype of the female physiological status?

The three olfactory secretomes of ewe, goat and pig yet studied show a specificity of the 2D-profile for a species, despite phylogenetically close primary sequences (Fig. 3) and common post-translational modifications [21]. The isoforms are dispatched in gels with a high specify from one species to another, and these profiles vary under hormonal control (this study, [21]). This specificity is reflected in different modalities of olfactory secretome changes in sexual activity season between sheep and goat. In ewe, our results show major differences between SR and SA olfactory secretome, for the three criteria investigated: the olfactory secretome profile obtained by 2D-E (a third string appears in SA), the olfactory secretome composition (apparition of SAL-like and VEG-like proteins in SA), and the post-translational modifications of the secretome (in SA: more phosphorylation, emergence of *O*-GlcNAcylation and N-glycosylation). These results put together indicate that the olfactory secretome becomes more complex when females are cycling (Additional file 1: Table S7), therefore is under endocrinological control. The complexification of the sensory equipment makes the ewe susceptible to detect a greater diversity of compounds, particularly emitted by a sexually active male, by the greater number of isoforms generated by newly secreted proteins and post-translational modifications. This suggests changes as well in the ram odour in SA, which is well-known to act as a primer pheromone, by inducing ovulation in the female [4–7]. In goats, only subtle variation of the olfactory secretome is observed. In this species, the differences between secretome profiles and composition are more important between females than between seasons, and the presence of post-translational modifications does not apparently vary, although precise identification of PTM sites are required to clarify this point. Whatever the modalities that remain to be determined in goat, they clearly differ from those evidenced in ewe, demonstrating that the same physiological status and associated behaviour could be governed by different molecular modalities on a common genetic background relative to OBPs. This is in accordance with the slight differences observed during the male effect: in ewe, the first ovulation is silent (i. e. not associated with heat behaviour) while in goat, females can express sexual receptivity already at the first ovulation [43, 44]. The quick response of the goats could indicate that the olfactive equipment needed to the reception of bulk odours is already present before the contact with the male, and in ewe that the delay between the male-female contact and the first ovulation corresponds to the expression of new olfactory proteins isoforms to potentiate the perception of the ram odours. This maturation could be under progesterone/oestradiol control. Moreover, the interspecific action of male odours could be explained by common isoforms expressed by the females of these two phylogenetically close species, tuned to the reception of common molecules, still unidentified, emitted by male sheep and goat.

## Conclusion

The olfactory secretome of ewe and goat constitutes a novel phenotype of the reproduction period, which could be helpful for the breeders to monitor females’ receptivity to the male effect. In this purpose, it is obvious that such an analysis of olfactory secretome cannot be performed easily by the methods used in this study. But Top-Down analysis in mass spectrometry should be considered, once the phenotypes have been determined.

## Methods

### Animals, and sample collection

Ewes (*Ovis aries*, Ile-de-France breed) and goats (*Capra hircus*, Alpine breed) were bred at INRA experimental farm (UEPAO, Nouzilly, France). Three ewes (30,056, 30,094, 30,118) and 3 goats (30,363, 30,422, 30,432) were followed for their physiological status by measure of progesterone concentration in blood by an immuno-enzymatic assay [45] (Additional file 1: Table S7). Nasal mucus was collected in a non-invasive manner, by gently wiping the nasal cavity with sterile gauzes, immediately put in 20 mL glass vials and stored at − 80 °C until protein extraction. Thus, samples from the same animal in sexual rest (SR) and in sexual activity (SA) seasons were compared, to limit the inter-individual variability frequently observed in Mammals. For amplification of cDNA sequences, respiratory mucosa was dissected from pentobarbital anaesthetized animals (one ewe and one goat) immediately after death and stored in RNAlater until RNA extraction.

### Protein analysis by electrophoresis

Chemicals and reagents were from Sigma-Aldrich, unless specified. Proteins were extracted from gauzes by phase partition with iced cooled solution of CHCl_3_/MeOH 2/1. Four mL were added to the gauzes and let stand at 4 °C during 1 h 30. Then, gauzes were squeezed with a syringe and 1 mL of MilliQ water. A second extraction was performed with 2 mL of CHCl_3_/MeOH and 0.5 mL of water. The resulting liquid solution was centrifuged at 4200 rpm during 20 min at 4 °C. The methanol phase (c. a. 4 mL) was collected, dried in Speed-Vac (Eppendorf) and stored at − 20 °C until use. Protein concentration was estimated in SDS-PAGE [46], in order to make standardization of aliquots to be further analysed by 2-dimensional electrophoresis (2-DE). For 2-DE, dried proteins were resuspended in 10 μL water, then in 120 μL of rehydration buffer as already described. Immobiline Dry Strips (IPG strips: pH 3–5.6, 7 cm, GE Healthcare or pH 4–7, 7 cm, Bio-Rad) were submitted to passive rehydration with samples during 16 h at room temperature (RT). First dimension (isoelectrofocalisation) was performed on PROTEAN i12™ IEF system (BioRad), with the standard program “7-cm Gradual S-1” (250 V rapid for 30 min, 1000 V gradual for 1 h, 5000 V gradual for 2 h and a hold of 5000 V), with a current limited to 50 μA/gel. When IEF was complete (9000 VH final), IPG strips were incubated successively for 15 min in the equilibration buffer (375 mM Tris-HCl pH 8.8, 6 M Urea, 2% (w/v) SDS and 30% (v/v) glycerol) containing first 1.5% (w/v) DTT then 2% (w/v) iodoacetamide. The second dimension was performed using 16.8% acrylamide SDS-PAGE in Mini-PROTEAN® Tetra cell (Bio-Rad) with the Prestained SDS-PAGE Standards Low Range or Precision Plus Protein Standard All blue or Precision Plus Protein Standard Dual Color or Precision Plus Protein Standard unstained (Bio-Rad) as molecular weight markers.

### Immunodetection of post-translational modifications

After 2-DE, gels were either stained with colloidal Coomassie blue R solution (12% trichloroacetic acid, 5% ethanolic solution of 0.035% Serva blue R-250) or transferred onto PVDF membranes (Mini-PVDF transfer pack, Bio-Rad) in the Trans-Blot Turbo Transfer system (Bio-Rad) according to manufacturer’s instructions. For immunodetection of *O*-GlcNAc modified proteins, membranes were blocked 1 h in 5% BSA PBS-T (Phosphate Buffered Saline, pH 7.2–0.05% Tween 20), then incubated 1 h at RT with CTD110.6 antibody (1:5000 dilution, Sigma-Aldrich) in blocking buffer. After six 5 min-washes, membranes were incubated 1 h at RT with secondary antibodies (1:30,000, anti-mouse IgM peroxidase conjugated, Sigma-Aldrich) in PBS-T. Signal was revealed by chemiluminescence with the SuperSignal™ West Dura Extended Duration Substrate (Pierce, Fisher Scientific). For GlcNAc competition assays, proteins were separated by SDS-PAGE, transferred onto PVDF membranes, which were blocked as above. Then, membranes were incubated 1 h (RT) with a pre-incubated mixture (1 h at RT) of CTD110.6 antibodies and 1 M GlcNAc (TCI) in TBS-T-5% BSA. They were washed and processed as above until ECL detection. Phosphorylated proteins were detected using anti-phospho-Threonine (Q7) or anti-phospho-Serine (Q5) antibodies (Qiagen) according to manufacturer’s protocol (Phosphoprotein Purification kit, Qiagen). The signal detection was performed as described above. Scanned images of stained gels and blots were merged using Image J software (NIH). To assess the specificity of Q5 antibodies, 15 μg of total proteins were digested by 20 U of Alkaline Phosphatase (Sigma-Aldrich) at 37 °C overnight in enzyme buffer (10 mM Tris, 5 mM MgCl_2_, 10 mM ZnCl_2_). An aliquot of the same sample (15 μg) was incubated at 37 °C overnight in the same buffer without alkaline phosphatase.

### Homology modelling

Three-dimensional structure of OBP2 and OBP4 proteins from both species were predicted by I-TASSER software, available at https://zhanglab.ccmb.med.umich.edu/I-TASSER/ [25]. For each OBP2 sequence, only one model was generated, with C-score of 0.84 (Oari-OBP2) and 0.87 (Chir-OBP2). Among the Top 10 identified structural analogues in the protein data bank (PDB) used to generate the model, the two OBP2 shared 31% of sequences with the dog lipocalin (PDB entry code 5X7Y, doi: 10.2210/pdb5X7Y/pdb), and 99% with the model superimposed with 5X7Y, at a root mean square deviation of 3.2 Å. For each OBP4, again only one model was generated with C-score of 1.03 (Oari-OBP4) and 1.08 (Chir-OBP4), with the allergen Bos d2 (from *Bos taurus*, PDB entry code 4WFV, doi: 10.2210/pdb4WFV/pdb) as best template (67% of identity with allergen Bos d2 and 97% with the model superimposed with AWFV, at a root mean square deviation of 2.7 Å).

### Identification of N-glycan chains

Fifteen μg of total proteins were solubilized in 20 μL of water, then incubated at 94 °C for 10 min. To remove N-glycan chains, proteins were digested with PNGase F (New England Biolabs®) with G7 buffer overnight at 37 °C, then the reaction was heat inactivated at 75 °C for 10 min. Digested and non-digested samples were analysed by SDS-PAGE. OBP N-glycan chains were immunodetected by lectin-blot. After separation by SDS-PAGE, proteins were transferred onto nitrocellulose membranes (Mini-nitrocellulose transfer pack, Bio-Rad) in the Trans-Blot Turbo Transfer system (Bio-Rad). Membranes were blocked 1 h 30 in 2% oxidized-BSA [47] in TBS (pH 7.4) buffer at RT. After three 5 min-washes, they were incubated 1 h 30 at RT with ConA-perox lectin (EY Laboratories, 1:1000 dilution) in 1 mM MgCl_2_, 1 mM CaCl_2_ in TBS buffer (pH 7.4). Membranes were washed three times during 10 min in TBS-T (TBS-0.1% Tween 20) and three times during 15 min in TBS buffer. The signal was revealed by chemiluminescence with the SuperSignal™ West Dura Extended Duration Substrate (Pierce, Fisher Scientific). RNase B (0.5 μg) was used as positive control.

### In silico search for *O. aries* and *C. hircus* OBP sequences

Putative OBP sequences were searched in goat genome, available at Ensembl database (not annotated), by performing a BlastX search (default parameters, Ensembl v. 87, Dec 2016) with porcine OBP, VEG and SAL nucleotide sequences as query. The sheep genome is partially annotated and available in GenBank database, we thus searched putative *O. aries* OBPs by BlastP (v. 2.6.0 Jan 2017) using porcine and bovine OBPs as query protein sequences (Table 3).

**Table 3:** Olfactory-binding protein sequences used in Blast searches for predicted ovine and caprine sequences

	Uniprot accession # for sheep OBP search	GenBank accession # for goat OBP search
Porcine OBP	P81245	NM_213796.1
Bovine OBP	P07435	XM_002700469.6
Porcine VEG	P53715	NM_213856.2
Rat VEG	P20289	NM_022945
Human VEG	P31025	NM_001252617
Porcine SAL	P81608	NM_213814.2

### Identification of proteins by mass spectrometry

Proteins separated by 2D-E were identified either by Matrix-Assisted Laser Desorption Ionization -Time Of Flight Mass Spectrometry (MALDI-TOF MS) or by nano-LC-nano-ESI-HCD-MS/MS-Orbitrap (nano-LC-MS/MS).

#### MALDI-TOF MS

The spots of interest were excised from the gel, destained with a mixture of 50% ACN/50 mM ammonium bicarbonate (v/v), then dehydrated with 100% ACN. Disulphide bridges were reduced with 10 mM DTT at 56 °C for 1 h, then alkylated with 50 mM iodoacetamide in 50 mM ammonium bicarbonate at RT for 45 min in the dark. Before trypsin digestion, spots were washed with 50 mM ammonium bicarbonate and dehydrated with 100% ACN. Spots were incubated overnight with 100 ng of Trypsin Gold (Promega) in 50 mM ammonium bicarbonate at 37 °C. Peptides were extracted from the gel by two incubations in 10% formic acid/45% ACN at 30 °C for 15 min, then with 5% formic acid/ 95% ACN at RT for 10 min. Peptides were desalted by centrifugation in Pierce® C18 spin column (Fisher Scientific) according to manufacturer’s instructions. Peptides were solubilized in 0.1% trifluoroacetic acid and mixed with 10 mg/mL α-Cyano-4-hydroxycinnamic acid matrix. MALDI-TOF MS analysis was performed on an Applied Biosystems, Voyager DE Pro mass spectrometer. The instrument was used in positive reflector mode, measuring peptide masses on a range of 500–4000 Da. Spectra were analysed with Data Explorer V4.6 (Applied Biosystems).

#### Nano-LC-MS/MS

Spots were excised from stained gels and analysed by the P3M platform of “Institut Pasteur de Lille” for trypsin digestion and mass spectrometry proteomics analysis. Digestion of in-gel proteins was performed as previously described [48]. An UltiMate 3000 RSLC nano System (Thermo Fisher Scientific) was used for the separation of protein digests. Peptides were automatically fractionated onto a commercial C18 reverse phase column (75 μm × 150 mm, 2-μm particle, PepMap100 RSLC column, Thermo Fisher Scientific) at 35 °C. Trapping was performed during 4 min at 5 μL/min, with solvent A (98% H_2_O, 2% acetonitrile-ACN and 0.1% formic acid-FA). Elution was performed using two solvents, A (0.1% FA in water) and B (0.1% FA in ACN) at a flow rate of 300 nL/min. Gradient separation was 2 min from 2 to 5% B, 12 min from 5 to 25% B, 2 min from 25 to 80% B, 3 min 80% B. The column was equilibrated for 8 min with 2% buffer B prior to the next sample analysis. The eluted peptides from the C18 column were analysed by a Q-Exactive device (Thermo Fisher Scientific). The electrospray voltage was 1.9 kV, and the capillary temperature was 275 °C. Full MS scans were acquired in the Orbitrap mass analyser over m/z 300–1200 range with a resolution of 35,000 (*m/z* 200). The target value was 5.00E + 05 and the maximum allowed ion accumulation times were 250 ms. Three most intense peaks with charge state between 2 and 4 were fragmented in the HCD collision cell with normalized collision energy of 35%, and tandem mass spectra were acquired in the Orbitrap mass analyser with a resolution of 17,500 at *m/z* 200. The target value was 5.00E+ 04 and the maximum allowed ion accumulation times were 150 ms. Dynamic exclusion was set to 7 s.

### Proteomic data analysis

Raw data collected during nano-LC-MS/MS analyses were processed and converted into *.mgf peak list format with Proteome Discoverer 1.4 (Thermo Fisher Scientific). MS/MS data were interpreted using search engine Mascot (version 2.4.0, Matrix Science, London, UK) installed on a local server. Searches were performed with a tolerance on mass measurement of 0.2 Da for the precursor and 0.02 Da for the ion fragment, against two composite target decoy databases built with *C. hircus* NCBI databases (TaxID = 9925, 05 January 2017, 46,032 entries) or *O. aries* UniProt database (TaxID: 9940, 20 March 2017, 27,139 entries), both fused with the sequences of recombinant trypsin and a list of classical contaminants (118 entries). Cysteine carbamidomethylation, methionine oxidation, protein N-terminal acetylation, cysteine propionamidation, serine and threonine phosphorylation were searched as variable modifications. Up to one trypsin missed cleavage was allowed. For each sample, peptides were filtered out according to the cut-off set for proteins hits with two or more peptides larger than nine residues, ion score > 15, no false positive protein was identified. The mass spectrometry proteomics data have been deposited to the ProteomeXchange Consortium via the PRIDE partner repository with the dataset identifier PXD014833 and 10.6019/PXD014833”.

### Amplification of the full-length nucleotide sequences of OBPs by rapid amplification of cDNA ends-polymerase chain reaction (RACE-PCR)

Total mRNA was extracted from 30 mg of nasal mucosa conserved in RNAlater (Qiagen) with the RNeasy Mini Kit (Qiagen). For amplification of the 5′ end, 5 μg of mRNA was first dephosphorylated with 10 U of CIAP (Calf Intestinal Alkaline Phosphatase, Invitrogen). Cap structures were removed with 5 U of RppH (5′ Pyrophosphohydrolase, New England BioLabs®), then mRNA was ligated to GeneRacer™RNA Oligo (GeneRacer™ kit, Invitrogen, 5′-CGACUGGAGCACGAGGACACUGACAUGGACUGAAGGAGUAGAAA-3′) with 5 U of T4 RNA ligase 1 (New England BioLabs®). Between each step, mRNA was cleaned up with the RNeasy MinElute CleanUp Kit (Qiagen). Capped mRNA (5’RACE) and native mRNA (3’RACE) were reverse transcribed using SuperScript™ IV RT kit (Invitrogen, Fisher Scientific) with 5′-gene specific primer (Additional file 1: Table S8) or GeneRacer™ Oligo dT primer (GeneRacer™ kit, Invitrogen), respectively. Amplifications were performed from 5 μL of RT template using Platinum™ *Taq* DNA Polymerase High Fidelity (Invitrogen) by touchdown PCR as follow: 94 °C for 2 min, followed by 5 cycles of 15 s at 94 °C and 30 s at 72 °C; 5 cycles of 15 s at 94 °C and 30 s at 70 °C; 25 cycles of 15 s at 94 °C, 30 s at 65 °C and 1 min at 68 °C. PCR products were cloned into pCR4®-TOPO vector (TOPO™-TA cloning™ kit, Invitrogen), then amplified into *Escherichia coli* One Shot™ Top10 chemically competent cells (Invitrogen). Recombinant plasmids were purified with QIAprep Spin Miniprep kit (Qiagen) and sequenced in both senses (Eurofins Genomics). Forward and reverse primers (**Table S8 in** Additional file 1) were designed from the sequences obtained by 5′- and 3′-RACE, in order to amplify the full-length sequences of the 2 major ovine and caprine OBPs. PCR amplification was performed from 5 μL of RT template with AccuPrime™ *Pfx* DNA Polymerase and Platinum™ *Taq* DNA Polymerase High Fidelity at a Tm of 65 °C, according to manufacturer’s protocol (Invitrogen). PCR products were cloned either into pCR®-Blunt II-TOPO or into pCR4®-TOPO vectors, respectively (Invitrogen). Recombinant plasmids were amplified, extracted and sequenced as described above.

### Molecular phylogenetic analysis by maximum likelihood method

The evolutionary history was inferred by using the Maximum Likelihood method based on the Tamura-Nei model [49]. The tree with the highest log likelihood (− 7216.11) was built. Initial tree(s) for the heuristic search were obtained automatically by applying Neighbour-Join and BioNJ algorithms to a matrix of pairwise distances estimated using the Maximum Composite Likelihood (MCL) approach, and then selecting the topology with superior log likelihood value. The tree was drawn to scale, with branch lengths measured in the number of substitutions per site. The analysis involved 23 nucleotide sequences. All positions containing gaps and missing data were eliminated. There were a total of 451 positions in the final dataset. Evolutionary analyses were conducted in MEGA7 [50].

## Supplementary information

**Additional file 1: Table S1.** Odorant-binding proteins identified in ewe 30,118 olfactory secretome in SR and SA by nano-LC-MS/MS. **Table S2.** Odorant-binding proteins identified in ewe 30,094 olfactory secretome in SR and SA by nano-LC-MS/MS. **Table S3.** Odorant-binding proteins identified in ewe 30,056 olfactory secretome in SR and SA by MALDI-TOF MS. **Table S4.** Odorant-binding proteins identified in goat 30,363 olfactory secretome in SR and SA by nano-LC-MS/MS or MALDI-TOF MS. **Table S5.** Odorant-binding proteins identified in goat 30,422 olfactory secretome in SR and SA by MALDI-TOF MS. **Table S6.** Odorant-binding proteins identified in goat 30,432 olfactory secretome in SR and SA by nano-LC-MS/MS. **Table S7.** Monitoring of progesterone concentration in blood of the ewes and goats used in this study**. Table S8.** Primers used for amplification of the major OBPs expressed in ewe and goat olfactory secretome. **Figure S1.** Sequence alignment of predicted lipocalins from sheep and goat genomes (BlastX searches). **Figure S2.** Two-dimensional electrophoresis of soluble proteins extracted from nasal mucus of ewes 30,094 and 30,056 in sexual rest (SR) and sexual activity (SA) periods. **Figure S3.** Two-dimensional electrophoresis of soluble proteins extracted from nasal mucus of goats 30,422 and 30,432 in sexual rest (SR) and sexual activity (SA). **Figure S4.** Full-length nucleotide and translated amino acid sequences of Chir-OBP2 and Chir-OBP4 obtained by RACE-PCR. **Figure S5.** Spot numbers labelled by anti-phosphoserine (a-d) and anti-O-GlcNAc (**e-g**) antibodies. **Figure S6.** Control of Q5 and CTD110.6 antibodies specificity. **Figure S7.** Immunodetection of phospho-threonine proteins by western-blot with Q7 Antibody (Qiagen). **Figure S8.** Comparison between naked and phosphorylated MS/MS spectra of the same peptide (THYIASSNTEK**T**GENGPFNVYLR).

## Data Availability

The datasets supporting the conclusions of this article are included within the article and its Additional file 1. The mass spectrometry proteomics data have been deposited to the ProteomeXchange Consortium via the PRIDE [24] partner repository with the dataset identifier PXD011371 and 10.6019/PXD01137. The full-length sequences of *Chir-OBP2*, *Chir-OBP4*, *Oari-OBP2*, and *Oari-OBP4* were deposited in GenBank database with accession numbers: MK908982, MK908983, MK908984 and MK908985, respectively.

## Abbreviations

2D-E: 2-dimensional electrophoresis
Chir: *Capra hircus*
CK: Casein kinase
EOGT: EGF-domain *O*-linked N-acetylglucosaminyl transferase
ESP: Exocrine gland secreted peptide
GnRH: Gonadotropin-releasing hormone
LH: Luteinizing hormone
MALDI-TOF MS: Matrix Assisted Laser Desorption Ionization - Time of Flight Mass Spectrometry
nano-LC-MS/MS: nano-LC-nano-ESI-HCD-MS/MS-Orbitrap
Oari: *Ovis aries*
OBP: Odorant-Binding Protein
*O*-GlcNAc: *O*-β-N-acetylglucosamine
*O*-GlcNAcylation: *O*-β-N-acetylglucosaminylation
RACE-PCR: Rapid amplification of cDNA-ends-polymerase chain reaction
SA: Sexual activity period
SAL: Salivary lipocalin
SR: Sexual rest period
VEG: Von Ebner’s gland protein
