# Supplementary material for: The olfactory secretome varies according to season in female sheep and goat
Source: BMC Genomics. 2019 Oct 30;20:794. doi: 10.1186/s12864-019-6194-z (PMC6822404; doi:10.1186/s12864-019-6194-z)

**The olfactory secretome varies according to season in female sheep and goat**

Paul CANN, Malika CHABI, Aliénor DELSART, Chrystelle LE DANVIC, Jean-Michel SALIOU, Manon CHASLES, Matthieu KELLER & Patricia NAGNAN-LE MEILLOUR

*BMC Genomics*

**Additional file 1**

**Table S1** Odorant-binding proteins identified in ewe 30118 olfactory secretome in SR and SA by nano-LC-MS/MS 03

**Table S2** Odorant-binding proteins identified in ewe 30094 olfactory secretome in SR and SA by nano-LC-MS/MS 04

**Table S3** Odorant-binding proteins identified in ewe 30056 olfactory secretome in SR and SA by MALDI-TOF MS 05

**Table S4** Odorant-binding proteins identified in goat 30363 olfactory secretome in SR and SA by nano-LC-MS/MS or MALDI-TOF MS 06

**Table S5** Odorant-binding proteins identified in goat 30422 olfactory secretome in SR and SA by MALDI-TOF MS 07

**Table S6** Odorant-binding proteins identified in goat 30432 olfactory secretome in SR and SA by nano-LC-MS/MS 10

**Table S7** Monitoring of progesterone concentration in blood of the ewes and goats used in this study 12

**Table S8** Primers used for amplification of the major OBPs expressed in ewe and goat olfactory secretome 13

**Figure S1** Sequence alignment of predicted lipocalins from sheep and goat genomes (BlastX searches) 14

**Figure S2** Two-dimensional electrophoresis of soluble proteins extracted from nasal mucus of ewes 30094 and 30056 in sexual rest (SR) and sexual activity (SA) periods 17

**Figure S3** Two-dimensional electrophoresis of soluble proteins extracted from nasal mucus of goats 30422 and 30432 in sexual rest (SR) and sexual activity (SA) periods 18

**Figure S4** Full-length nucleotide and translated amino acid sequences of Chir-OBP2 and Chir-OBP4 obtained by RACE-PCR 19

**Figure S5** Spot numbers labelled by anti-phosphoserine (a-d) and anti-*O*-GlcNAc (e-g) antibodies. 20

**Figure S6** Control of Q5 and CTD110.6 antibodies specificity 21

**Figure S7** Immunodetection of phospho-threonine proteins by western-blot with Q7 Antibody (Qiagen) 22

**Figure S8** Comparison between naked and phosphorylated MS/MS spectra of the same peptide (THYIASSNTEK**T**GENGPFNVYLR) 23

**Table S1** Odorant-binding proteins identified in ewe 30118 olfactory secretome in SR (spots 1 to 10, corresponding to Fig. 1a) and SA (spots 11 to 34, corresponding to Fig. 1b) by nano-LC-MS/MS.

| Spot No. | Protein identification | UniProt KB accession number | % coverage (Peptides matched) | Number of spectra | Spot No. | Protein identification | UniProt KB accession number | % coverage (Peptides matched) | Number of spectra |
| --- | --- | --- | --- | --- | --- | --- | --- | --- | --- |
| 1 | Oari-OBP2 | W5PGN0 | 34.1 (5) | 24 | 16 | * |  |  |  |
|  | Oari-OBP4 | W5PHS2 | 30.9 (4) | 6 | 17 | Oari-OBP2 | W5PGN0 | 14.3 (2) | 4 |
| 2 | Oari-OBP2 | W5PGN0 | 18.7 (2) | 10 |  | Oari-OBP4 | W5PHS2 | 12.1 (2) | 4 |
| 3 | Oari-OBP2 | W5PGN0 | 18.7 (2) | 4 | 18 | Oari-OBP2 | W5PGN0 | 14.3 (2) | 4 |
| 4 | Oari-OBP2 | W5PGN0 | 18.7 (2) | 4 |  | Oari-OBP3 | W5PGW3 | 8.9 (3) | 4 |
| 5 | * |  |  |  |  | Oari-OBP4 | W5PHS2 | 12.1 (2) | 3 |
| 6 | * |  |  |  | 19 | Oari-OBP3 | W5PGW3 | 8.9 (2) | 2 |
| 7 | * |  |  |  | 20 | Oari-OBP3 | W5PGW3 | 15.6 (4) | 7 |
| 8 | Oari-OBP2 | W5PGN0 | 26.4 (4) | 11 | 21 | Oari-OBP3 | W5PGW3 | 8.9 (3) | 7 |
|  | Oari-OBP4 | W5PHS2 | 30.9 (4) | 44 | 22 | * |  |  |  |
| 9 | Oari-OBP2 | W5PGN0 | 35.2 (5) | 48 | 23 | * |  |  |  |
|  | Oari-OBP4 | W5PHS2 | 18.7 (3) | 11 | 24 | Oari-OBP2 | W5PGN0 | 26.4 (4) | 14 |
| 10 | Oari-OBP2 | W5PGN0 | 35.2 (6) | 60 |  | Oari-OBP4 | W5PHS2 | 12.1 (3) | 16 |
| 11 | Oari-OBP2 | W5PGN0 | 26.4 (3) | 6 | 25 | Oari-OBP1 | W5PHM2 | 34.1 (3) | 23 |
|  | Oari-OBP4 | W5PHS2 | 12.1 (2) | 2 |  | Oari-OBP2 | W5PGN0 | 30.9 (5) | 26 |
|  | Oari-SAL1 | W5P8W4 | 18.5 (3) | 80 |  | Oari-OBP4 | W5PHS2 | 7.6 (6) | 3 |
|  | Oari-SAL2 | W5P8Y1 | 27.2 (4) | 48 | 26 | Oari-OBP2 | W5PGN0 | 35.2 (6) | 28 |
| 12 | Oari-OBP4 | W5PHS2 | 12.1 (2) | 9 |  | Oari-OBP4 | W5PHS2 | 25.4 (5) | 45 |
|  | Oari-SAL1 | W5P8W4 | 13.1 (2) | 53 | 27 | Oari-OBP2 | W5PGN0 | 48.5 (8) | 89 |
|  | Oari-SAL2 | W5P8Y1 | 21.8 (3) | 35 |  | Oari-OBP4 | W5PHS2 | 18.7 (3) | 5 |
| 13 | Oari-SAL1 | W5P8W4 | 18.5 (3) | 68 | 28 | Oari-OBP2 | W5PGN0 | 38.5 (7) | 51 |
|  | Oari-SAL2 | W5P8Y1 | 18.5 (3) | 35 |  | Oari-OBP4 | W5PHS2 | 12.1 (2) | 4 |
| 14 | Oari-SAL1 | W5P8W4 | 13.1 (2) | 14 | 29 | Oari-OBP2 | W5PGN0 | 34.1 (5) | 28 |
|  | Oari-SAL2 | W5P8Y1 | 13.1 (2) | 8 | 30 | * |  |  |  |
| 15 | Oari-OBP2 | W5PGN0 | 34.1 (5) | 43 | 31 | * |  |  |  |
|  | Oari-OBP4 | W5PHS2 | 12.1 (2) | 5 | 32 | * |  |  |  |
|  | Oari-SAL1 | W5P8W4 | 18.5 (3) | 28 | 33 | * |  |  |  |
|  | Oari-SAL2 | W5P8Y1 | 18.5 (3) | 13 | 34 | * |  |  |  |

*No OBP identified in these spots

**Table S2** Odorant-binding proteins identified in ewe 30094 olfactory secretome in SR (spots 35 to 48, corresponding to Supplementary Fig. S2a) and SA (spots 49 to 71, corresponding to Supplementary Fig. S2b) by nano-LC-MS/MS.

| Spot No. | Protein identification | UniProt KB accession number | % coverage (Peptides matched) | Number of spectra | Spot No. | Protein identification | UniProt KB accession number | % coverage (Peptides matched) | Number of spectra |
| --- | --- | --- | --- | --- | --- | --- | --- | --- | --- |
| 35 | Oari-OBP2 | W5PGN0 | 14.3 (2) | 6 | 56 | Oari-OBP2 | W5PGN0 | 34.1 (5) | 23 |
|  | Oari-OBP4 | W5PHS2 | 12.1 (2) | 2 |  | Oari-OBP4 | W5PHS2 | 18.7 (3) | 11 |
| 36 | Oari-OBP2 | W5PGN0 | 26.4 (3) | 7 |  | Oari-SAL1 | W5P8W4 | 38.1 (3) | 12 |
| 37 | Oari-OBP2 | W5PGN0 | 14.3 (2) | 5 |  | Oari-SAL2 | W5P8Y1 | 18.5 (2) | 3 |
| 38 | Oari-OBP2 | W5PGN0 | 26.4 (3) | 5 | 57 | * |  |  |  |
| 39 | * |  |  |  | 58 | Oari-OBP2 | W5PGN0 | 26.4 (3) | 7 |
| 40 | * |  |  |  |  | Oari-OBP4 | W5PHS2 | 12.1 (2) | 2 |
| 41 | * |  |  |  |  | Oari-SAL1 | W5P8W4 | 38.1 (3) | 18 |
| 42 | * |  |  |  |  | Oari-SAL2 | W5P8Y1 | 18.5 (2) | 4 |
| 43 | * |  |  |  | 59 | Oari-OBP2 | W5PGN0 | 18.7 (2) | 3 |
| 44 | * |  |  |  | 60 | Oari-OBP2 | W5PGN0 | 18.7 (2) | 4 |
| 45 | Oari-OBP1 | W5PHM2 | 28.7 (3) | 5 |  | Oari-OBP3 | W5PGW3 | 6.7 (2) | 4 |
|  | Oari-OBP2 | W5PGN0 | 34.1 (5) | 22 |  | Oari-SAL2 | W5P8Y1 | 16.3 (2) | 2 |
|  | Oari-OBP4 | W5PHS2 | 30.9 (4) | 19 | 61 | Oari-OBP2 | W5PGN0 | 34.1 (5) | 67 |
| 46 | Oari-OBP2 | W5PGN0 | 48.5 (8) | 75 |  | Oari-OBP4 | W5PHS2 | 12.1 (2) | 20 |
|  | Oari-OBP4 | W5PHS2 | 30.9 (6) | 19 | 62 | Oari-OBP2 | W5PGN0 | 44.1 (8) | 81 |
| 47 | Oari-OBP2 | W5PGN0 | 38.5 (5) | 34 |  | Oari-OBP4 | W5PHS2 | 18.7 (5) | 41 |
| 48 | Oari-OBP2 | W5PGN0 | 18.7 (2) | 3 | 63 | Oari-OBP2 | W5PGN0 | 35.2 (7) | 116 |
| 49 | Oari-OBP2 | W5PGN0 | 34.1 (5) | 27 |  | Oari-OBP4 | W5PHS2 | 18.7 (3) | 3 |
|  | Oari-SAL1 | W5P8W4 | 38.1 (5) | 62 | 64 | Oari-OBP2 | W5PGN0 | 44.1 (8) | 77 |
|  | Oari-SAL2 | W5P8Y1 | 27.2 (4) | 35 |  | Oari-OBP4 | W5PHS2 | 25.4 (6) | 19 |
| 50 | Oari-OBP2 | W5PGN0 | 34.1 (5) | 12 | 65 | Oari-OBP2 | W5PGN0 | 44.1 (8) | 70 |
|  | Oari-SAL1 | W5P8W4 | 38.1 (4) | 51 |  | Oari-OBP4 | W5PHS2 | 18.7 (3) | 8 |
|  | Oari-SAL2 | W5P8Y1 | 27.2 (4) | 26 | 66 | Oari-OBP2 | W5PGN0 | 35.2 (7) | 109 |
| 51 | Oari-OBP2 | W5PGN0 | 18.7 (2) | 3 |  | Oari-OBP4 | W5PHS2 | 18.7 (3) | 7 |
|  | Oari-OBP4 | W5PHS2 | 18.7 (3) | 7 | 67 | Oari-OBP2 | W5PGN0 | 44.1 (7) | 46 |
|  | Oari-SAL1 | W5P8W4 | 38.1 (5) | 62 |  | Oari-OBP4 | W5PHS2 | 25.4 (5) | 47 |
|  | Oari-SAL2 | W5P8Y1 | 27.2 (4) | 31 | 68 | Oari-OBP2 | W5PGN0 | 35.2 (7) | 115 |
| 52 | Oari-SAL1 | W5P8W4 | 45.8 (9) | 59 |  | Oari-OBP4 | W5PHS2 | 13.2 (2) | 2 |
|  | Oari-SAL2 | W5P8Y1 | 33.8 (5) | 25 | 69 | Oari-VEG1 | W5NUS5 | 73.1 (8) | 35 |
| 53 | Oari-OBP2 | W5PGN0 | 15.4 (2) | 3 | 70 | Oari-OBP2 | W5PGN0 | 18.7 (2) | 3 |
|  | Oari-SAL1 | W5P8W4 | 40.3 (6) | 50 |  | Oari-VEG1 | W5NUS5 | 61 (8) | 22 |
|  | Oari-SAL2 | W5P8Y1 | 27.2 (4) | 22 | 71 | Oari-OBP2 | W5PGN0 | 26.4 (3) | 5 |
| 54 | Oari-OBP2 | W5PGN0 | 34.1 (6) | 49 |  | Oari-OBP4 | W5PHS2 | 12.1 (2) | 2 |
| 55 | Oari-OBP2 | W5PGN0 | 43.1 (6) | 49 |  |  |  |  |  |
|  | Oari-OBP4 | W5PHS2 | 12.1 (2) | 3 |  |  |  |  |  |
|  | Oari-SAL1 | W5P8W4 | 32.4 (3**)** | 8 |  |  |  |  |  |
|  | Oari-SAL2 | W5P8Y1 | 13.2 (2) | 3 |  |  |  |  |  |

*No OBP identified in these spots

**Table S3** Odorant-binding proteins identified in ewe 30056 olfactory secretome in SR (spots 72 to 83, corresponding to Supplementary Fig. S2c) and SA (spots 84 to 103, corresponding to Supplementary Fig. S2d) by MALDI-TOF MS.

| Spot No. | Protein identification | UniProt KB accession number | % coverage | Number matched peptides | Spot No. | Protein identification | UniProt KB accession number | % coverage | Number matched peptides |
| --- | --- | --- | --- | --- | --- | --- | --- | --- | --- |
| 72 | Oari-OBP2 | W5PGN0 | 31.6 | 7 | 86 | Oari-OBP2 | W5PGN0 | 20.9 | 3 |
|  | Oari-OBP3 | W5PGW3 | 27.9 | 3 |  | Oari-SAL1/2 | W5P8Y1/W5P8W4 | 34.1 | 7 |
|  | Oari-OBP4 | W5PHS2 | 12.2 | 2 | 87 | Oari-OBP3 | W5PGW3 | 4.5 | 2 |
| 73 | Oari-OBP2 | W5PGN0 | 15.8 | 2 |  | Oari-SAL1/2 | W5P8Y1/W5P8W4 | 34.1 | 7 |
|  | Oari-OBP4 | W5PHS2 | 14.7 | 2 | 88 | Oari-OBP1 | W5PHM2 | 11.6 | 2 |
| 74 | Oari-OBP4 | W5PHS2 | 14.7 | 3 |  | Oari-OBP3 | W5PGW3 | 4.5 | 2 |
| 75 | Oari-SAL1/2 | W5P8Y1/W5P8W4 | 9 | 2 |  | Oari-SAL1/2 | W5P8Y1/W5P8W4 | 23.4 | 4 |
|  | Oari-OBP4 | W5PHS2 | 6.4 | 2 | 89 | Oari-OBP1 | W5PHM2 | 13.5 | 2 |
| 76 | Oari-OBP2 | W5PGN0 | 15.2 | 2 |  | Oari-OBP2 | W5PGN0 | 37.3 | 5 |
|  | Oari-OBP4 | W5PHS2 | 14.7 | 3 |  | Oari-SAL1/2 | W5P8Y1/W5P8W4 | 15 | 2 |
| 77 | Oari-OBP2 | W5PGN0 | 15.2 | 2 | 90 | Oari-OBP1 | W5PHM2 | 12.9 | 2 |
|  | Oari-OBP4 | W5PHS2 | 11.5 | 2 | 91 | Oari-SAL1/2 | W5P8Y1/W5P8W4 | 15.6 | 2 |
| 78 | Oari-OBP2 | W5PGN0 | 31.6 | 7 | 92 | Oari-OBP1 | W5PHM2 | 11.6 | 2 |
|  | Oari-OBP3 | W5PGW3 | 20.8 | 2 | 93 | Oari-OBP1 | W5PHM2 | 20 | 3 |
|  | Oari-OBP4 | W5PHS2 | 19.9 | 4 |  | Oari-OBP2 | W5PGN0 | 15.8 | 2 |
| 79 | Oari-OBP2 | W5PGN0 | 31.6 | 8 |  | Oari-OBP4 | W5PHS2 | 10.9 | 2 |
|  | Oari-OBP3 | W5PGW3 | 20.8 | 2 | 94 | Oari-OBP2 | W5PGN0 | 23.4 | 3 |
|  | Oari-OBP4 | W5PHS2 | 23.7 | 3 | 95 | Oari-OBP2 | W5PGN0 | 30.4 | 5 |
| 80 | Oari-OBP2 | W5PGN0 | 24.7 | 4 |  | Oari-OBP3 | W5PGW3 | 20.8 | 2 |
|  | Oari-OBP3 | W5PGW3 | 20.8 | 2 | 96 | Oari-OBP2 | W5PGN0 | 25.3 | 5 |
|  | Oari-OBP4 | W5PHS2 | 10.3 | 2 | 97 | Oari-OBP2 | W5PGN0 | 17.1 | 2 |
| 81 | Oari-OBP2 | W5PGN0 | 31.6 | 7 | 98 | Oari-OBP2 | W5PGN0 | 30.4 | 5 |
|  | Oari-OBP3 | W5PGW3 | 20.8 | 2 |  | Oari-OBP3 | W5PGW3 | 20.8 | 2 |
|  | Oari-SAL1/2 | W5P8Y1/W5P8W4 | 10.2 | 2 |  | Oari-OBP4 | W5PHS2 | 16 | 2 |
|  | Oari-OBP4 | W5PHS2 | 10.3 | 2 | 99 | Oari-OBP2 | W5PGN0 | 43.7 | 7 |
| 82 | Oari-OBP2 | W5PGN0 | 31.6 | 8 |  | Oari-OBP3 | W5PGW3 | 20.8 | 2 |
|  | Oari-OBP3 | W5PGW3 | 20.8 | 2 |  | Oari-OBP4 | W5PHS2 | 21.2 | 4 |
|  | Oari-OBP4 | W5PHS2 | 10.3 | 2 |  | Oari-SAL1/2 | W5P8Y1/W5P8W4 | 15 | 2 |
| 83 | Oari-OBP2 | W5PGN0 | 31.6 | 6 | 100 | Oari-OBP2 | W5PGN0 | 23.4 | 5 |
|  | Oari-OBP3 | W5PGW3 | 20.8 | 3 |  | Oari-OBP4 | W5PHS2 | 17.3 | 2 |
|  | Oari-OBP4 | W5PHS2 | 10.3 | 2 | 101 | Oari-OBP2 | W5PGN0 | 50.6 | 9 |
| 84 | Oari-OBP2 | W5PGN0 | 15.8 | 2 |  | Oari-OBP3 | W5PGW3 | 20.8 | 2 |
|  | Oari-SAL1/2 | W5P8Y1/W5P8W4 | 34.1 | 7 | 102 | Oari-OBP2 | W5PGN0 | 20.9 | 3 |
| 85 | Oari-OBP2 | W5PGN0 | 18.4 | 2 | 103 | Oari-OBP2 | W5PGN0 | 11.4 | 2 |
|  | Oari-SAL1/2 | W5P8Y1/W5P8W4 | 34.1 | 7 |  |  |  |  |  |

*No OBP identified in these spots

**Table S4** Odorant-binding proteins identified in goat 30363 olfactory secretome in SR (spots 1 to 26, corresponding to Fig. 1c) and SA (spots 27 to 45, corresponding to Fig. 1d) by nano-LC-MS/MS for SR and MALDI-TOF MS for SA.

| Spot N° | Protein identification | UniProt KB accession number | % coverage (Peptides matched) | Number of spectra | Spot N° | Protein identification | UniProt KB accession number | % coverage | Number matched peptides |
| --- | --- | --- | --- | --- | --- | --- | --- | --- | --- |
| 1 | Chir-OBP2 | XP_017899208.1 | 43.1 (7) | 55 | 30 | Chir-OBP3 | XP_005701296.2 | 9.6 | 2 |
|  | Chir-OBP4 | XP_017899515.1 | 30.8 (5) | 25 |  | Chir-OBP5 | XP_017899538.1 | 16.5 | 2 |
|  | Chir-OBP6 | XP_017900101.1 | 17.1 (2) | 2 |  | Chir-OBP6 | XP_017900101.1 | 12.5 | 2 |
| 2 | Chir-OBP2 | XP_017899208.1 | 34.2 (4) | 19 |  | Chir-VEG1 | XP_005687416.1 | 15.9 | 2 |
|  | Chir-OBP4 | XP_017899515.1 | 13.5 (3) | 3 | 31 | Chir-OBP2 | XP_017899208.1 | 37.3 | 6 |
|  | Chir-SAL1 | XP_017908099.1 | 29.3 (5) | 7 |  | Chir-OBP3 | XP_005701296.2 | 24.8 | 3 |
| 3 | Chir-OBP4 | XP_017899515.1 | 25.7 (4) | 19 |  | Chir-OBP4 | XP_017899515.1 | 18.6 | 3 |
| 4 | * |  |  |  |  | Chir-OBP6 | XP_017900101.1 | 16.5 | 2 |
| 5 | Chir-OBP2 | XP_017899208.1 | 43.1 (8) | 54 |  | Chir-SAL1/2 | XP_0170809(8/9).1 | 17.4 | 2 |
|  | Chir-OBP4 | XP_017899515.1 | 37.2 (6) | 57 | 32 | Chir-OBP2 | XP_017899208.1 | 41.8 | 6 |
| 6 | Chir-OBP2 | XP_017899208.1 | 43.1 (8) | 74 |  | Chir-OBP3 | XP_005701296.2 | 24.8 | 3 |
|  | Chir-OBP4 | XP_017899515.1 | 37.2 (6) | 49 |  | Chir-OBP4 | XP_017899515.1 | 10.9 | 2 |
| 7 | Chir-OBP2 | XP_017899208.1 | 52.5 (9) | 103 |  | Chir-OBP5 | XP_017899538.1 | 17.1 | 3 |
|  | Chir-OBP4 | XP_017899515.1 | 18.6 (4) | 13 |  | Chir-SAL1/2 | XP_0170809(8/9).1 | 23.4 | 3 |
| 8 | Chir-OBP2 | XP_017899208.1 | 43.1 (8) | 108 |  | Chir-VEG2 | XP_017911671.1 | 21.6 | 2 |
|  | Chir-OBP4 | XP_017899515.1 | 30.8 (5) | 12 |  | Chir-VEG3 | XP_017910286.1 | 20.9 | 2 |
| 9 | Chir-OBP2 | XP_017899208.1 | 52.5 (9) | 119 | 33 | Chir-OBP2 | XP_017899208.1 | 50.0 | 10 |
|  | Chir-OBP4 | XP_017899515.1 | 12.1 (2) | 6 |  | Chir-OBP4 | XP_017899515.1 | 10.9 | 2 |
| 10 | Chir-OBP2 | XP_017899208.1 | 52.5 (9) | 88 | 34 | Chir-OBP2 | XP_017899208.1 | 48.1 | 10 |
|  | Chir-OBP4 | XP_017899515.1 | 18.6 (4) | 10 |  | Chir-OBP4 | XP_017899515.1 | 40.4 | 5 |
|  | Chir-VEG1 | XP_005687416.1 | 28.6 (3) | 4 |  | Chir-VEG1 | XP_005687416.1 | 19.1 | 2 |
| 11 | Chir-OBP2 | XP_017899208.1 | 52.5 (9) | 75 | 35 | Chir-OBP2 | XP_017899208.1 | 52.2 | 9 |
|  | Chir-OBP4 | XP_017899515.1 | 30.8 (5) | 32 |  | Chir-OBP4 | XP_017899515.1 | 21.2 | 3 |
|  | Chir-VEG2 | XP_017911671.1 | 22.8 (2) | 4 |  | Chir-SAL1/2 | XP_0170809(8/9).1 | 17.4 | 2 |
| 12 | Chir-OBP2 | XP_017899208.1 | 43.1 (8) | 54 |  | Chir-VEG2 | XP_017911671.1 | 28.5 | 3 |
|  | Chir-OBP4 | XP_017899515.1 | 37.2 (6) | 21 | 36 | Chir-OBP2 | XP_017899208.1 | 30.4 | 3 |
| 13 | Chir-OBP2 | XP_017899208.1 | 67.7 (8) | 63 |  | Chir-OBP6 | XP_017900101.1 | 12.5 | 2 |
|  | Chir-OBP4 | XP_017899515.1 | 30.8 (5) | 12 | 37 | Chir-OBP2 | XP_017899208.1 | 50.0 | 5 |
| 14 | Chir-OBP2 | XP_017899208.1 | 43.1 (7) | 46 |  | Chir-OBP6 | XP_017900101.1 | 21.7 | 3 |
|  | Chir-OBP4 | XP_017899515.1 | 30.8 (5) | 10 | 38 | Chir-OBP2 | XP_017899208.1 | 45.6 | 7 |
| 15 | Chir-OBP2 | XP_017899208.1 | 34.2 (6) | 50 |  | Chir-OBP4 | XP_017899515.1 | 10.3 | 2 |
|  | Chir-OBP4 | XP_017899515.1 | 24.4 (3) | 7 |  | Chir-VEG3 | XP_017910286.1 | 16.3 | 2 |
| 16 | * |  |  |  | 39 | Chir-OBP2 | XP_017899208.1 | 30.4 | 4 |
| 17 | Chir-OBP2 | XP_017899208.1 | 26 (2) | 3 |  | Chir-VEG2 | XP_017911671.1 | 21.6 | 2 |
|  | Chir-OBP4 | XP_017899515.1 | 19.2 (2) | 2 | 40 | Chir-OBP2 | XP_017899208.1 | 48.1 | 6 |
| 18 | Chir-OBP4 | XP_017899515.1 | 19.2 (2) | 2 |  | Chir-OBP4 | XP_017899515.1 | 23.1 | 2 |
| 19 | Chir-OBP4 | XP_017899515.1 | 19.2 (2) | 5 |  | Chir-VEG2 | XP_017911671.1 | 21.6 | 2 |
| 20 | Chir-OBP2 | XP_017899208.1 | 27.2 (3) | 16 | 41 | Chir-OBP2 | XP_017899208.1 | 50.0 | 8 |
| 21 | * |  |  |  |  | Chir-OBP4 | XP_017899515.1 | 16.0 | 3 |
| 22 | Chir-OBP2 | XP_017899208.1 | 14.6 (2) | 4 |  | Chir-SAL1/2 | XP_0170809(8/9).1 | 17.4 | 2 |
| 23 | * |  |  |  |  | Chir-VEG2 | XP_017911671.1 | 32.1 | 5 |
| 24 | Chir-OBP2 | XP_017899208.1 | 22.8 (4) | 35 | 42 | Chir-OBP2 | XP_017899208.1 | 15.8 | 2 |
| 25 | Chir-OBP2 | XP_017899208.1 | 22.8 (4) | 5 |  | Chir-VEG2 | XP_017911671.1 | 19.1 | 3 |
| 26 | * |  |  |  | 43 | Chir-OBP2 | XP_017899208.1 | 24.7 | 3 |
| 27 | Chir-OBP2 | XP_017899208.1 | 30.4 | 3 |  | Chir-SAL1/2 | XP_0170809(8/9).1 | 18.6 | 2 |
| 28 | Chir-OBP2 | XP_017899208.1 | 58.9 | 8 |  | Chir-VEG2 | XP_017911671.1 | 19.1 | 2 |
|  | Chir-OBP5 | XP_017899538.1 | 9.2 | 2 | 44 | Chir-SAL1/2 | XP_0170809(8/9).1 | 18.6 | 2 |
|  | Chir-OBP6 | XP_017900101.1 | 9.2 | 2 | 45 | Chir-OBP2 | XP_017899208.1 | 15.8 | 2 |
| 29 | Chir-OBP2 | XP_017899208.1 | 24.7 | 5 |  | Chir-OBP4 | XP_017899515.1 | 18.6 | 2 |
|  | Chir-OBP6 | XP_017900101.1 | 17.1 | 3 |  | Chir-OBP6 | XP_017900101.1 | 21.7 | 3 |

*No OBP identified in these spots

**Table S5** Odorant-binding proteins identified in goat 30422 olfactory secretome in SR (spots 46 to 73, corresponding to Supplementary Fig. S3a) and SA (spots 74 to 82, corresponding to Supplementary Fig. S3b) by MALDI-TOF MS.

| Spot N° | Protein identification | UniProt KB accession number | % coverage | Number matched peptides | Spot N° | Protein identification | UniProt KB accession number | % coverage | Number matched peptides |
| --- | --- | --- | --- | --- | --- | --- | --- | --- | --- |
| 46 | Chir-OBP2 | XP_017899208.1 | 50 | 7 | 61 | Chir-OBP2 | XP_017899208.1 | 50 | 10 |
|  | Chir-OBP4 | XP_017899515.1 | 24.4 | 3 |  | Chir-OBP4 | XP_017899515.1 | 22.4 | 4 |
|  | Chir-OBP5 | XP_017899538.1 | 9.2 | 2 |  | Chir-SAL1/2 | XP_0170809(8/9).1 | 21.6 | 3 |
|  | Chir-SAL1 | XP_017908099.1 | 27 | 3 |  | Chir-VEG2 | XP_017911671.1 | 27.2 | 3 |
|  | Chir-SAL2 | XP_017908098.1 | 17.4 | 2 | 62 | Chir-OBP2 | XP_017899208.1 | 50 | 11 |
|  | Chir-VEG2 | XP_017911671.1 | 39.5 | 4 |  | Chir-OBP4 | XP_017899515.1 | 18.6 | 3 |
| 47 | Chir-OBP2 | XP_017899208.1 | 23.4 | 4 |  | Chir-SAL1 | XP_017908099.1 | 15 | 2 |
|  | Chir-OBP3 | XP_005701296.2 | 15.9 | 3 |  | Chir-SAL2 | XP_017908098.1 | 24 | 3 |
|  | Chir-OBP4 | XP_017899515.1 | 12.2 | 2 |  | Chir-VEG2 | XP_017911671.1 | 21.6 | 3 |
|  | Chir-OBP5 | XP_017899538.1 | 9.2 | 2 | 63 | Chir-OBP2 | XP_017899208.1 | 50 | 10 |
|  | Chir-OBP6 | XP_017900101.1 | 16.5 | 2 |  | Chir-OBP4 | XP_017899515.1 | 22.4 | 4 |
|  | Chir-SAL1/2 | XP_0170809(8/9).1 | 17.4 | 2 |  | Chir-SAL1 | XP_017908099.1 | 15 | 2 |
|  | Chir-VEG2 | XP_017911671.1 | 23.5 | 3 |  | Chir-SAL2 | XP_017908098.1 | 24 | 3 |
| 48 | Chir-OBP2 | XP_017899208.1 | 43 | 8 |  | Chir-VEG1 | XP_005687416.1 | 21 | 2 |
|  | Chir-OBP3 | XP_005701296.2 | 14 | 3 |  | Chir-VEG2 | XP_017911671.1 | 21.6 | 3 |
|  | Chir-OBP4 | XP_017899515.1 | 10.3 | 2 | 64 | Chir-OBP2 | XP_017899208.1 | 50 | 10 |
|  | Chir-SAL1/2 | XP_0170809(8/9).1 | 16.8 | 2 |  | Chir-OBP3 | XP_005701296.2 | 18.5 | 2 |
|  | Chir-VEG2 | XP_017911671.1 | 25.9 | 3 |  | Chir-OBP5 | XP_017899538.1 | 9.2 | 3 |
| 49 | Chir-OBP2 | XP_017899208.1 | 25.3 | 3 |  | Chir-SAL1/2 | XP_0170809(8/9).1 | 21.6 | 3 |
|  | Chir-OBP3 | XP_005701296.2 | 16.6 | 2 |  | Chir-VEG2 | XP_017911671.1 | 23.5 | 3 |
|  | Chir-OBP6 | XP_017900101.1 | 7.2 | 2 | 65 | Chir-OBP4 | XP_017899515.1 | 17.3 | 2 |
|  | Chir-VEG2 | XP_017911671.1 | 21.6 | 2 |  | Chir-SAL1/2 | XP_0170809(8/9).1 | 18.6 | 3 |
| 50 | Chir-OBP2 | XP_017899208.1 | 38.6 | 6 |  | Chir-VEG2 | XP_017911671.1 | 23.5 | 2 |
|  | Chir-OBP5 | XP_017899538.1 | 9.2 | 3 | 66 | Chir-OBP2 | XP_017899208.1 | 17.1 | 3 |
|  | Chir-SAL1/2 | XP_0170809(8/9).1 | 17.4 | 2 |  | Chir-OBP3 | XP_005701296.2 | 22.9 | 3 |
|  | Chir-VEG2 | XP_017911671.1 | 25.9 | 3 |  | Chir-SAL1/2 | XP_0170809(8/9).1 | 21 | 3 |
| 51 | Chir-OBP2 | XP_017899208.1 | 48.1 | 9 |  | Chir-VEG2 | XP_017911671.1 | 30.3 | 3 |
|  | Chir-OBP3 | XP_005701296.2 | 28.7 | 4 | 67 | Chir-OBP2 | XP_017899208.1 | 20.3 | 2 |
|  | Chir-OBP4 | XP_017899515.1 | 16 | 3 |  | Chir-OBP3 | XP_005701296.2 | 22.9 | 3 |
|  | Chir-SAL1/2 | XP_0170809(8/9).1 | 24.6 | 4 |  | Chir-SAL1/2 | XP_0170809(8/9).1 | 23.4 | 3 |
|  | Chir-VEG2 | XP_017911671.1 | 25.9 | 3 |  | Chir-VEG2 | XP_017911671.1 | 29 | 4 |
| 52 | Chir-OBP2 | XP_017899208.1 | 50 | 10 | 68 | Chir-OBP2 | XP_017899208.1 | 24.1 | 3 |
|  | Chir-OBP3 | XP_005701296.2 | 21.7 | 3 |  | Chir-OBP5 | XP_017899538.1 | 9.2 | 2 |
|  | Chir-OBP4 | XP_017899515.1 | 26.9 | 5 | 69 | Chir-OBP2 | XP_017899208.1 | 19.6 | 2 |
|  | Chir-OBP5 | XP_017899538.1 | 5.3 | 2 |  | Chir-SAL1/2 | XP_0170809(8/9).1 | 13.8 | 2 |
|  | Chir-SAL1/2 | XP_0170809(8/9).1 | 24.6 | 4 |  | Chir-VEG2 | XP_017911671.1 | 23.5 | 4 |
|  | Chir-VEG2 | XP_017911671.1 | 21.6 | 2 | 70 | Chir-OBP2 | XP_017899208.1 | 19.6 | 2 |
| 53 | Chir-OBP2 | XP_017899208.1 | 48.1 | 9 |  | Chir-OBP5 | XP_017899538.1 | 9.2 | 2 |
|  | Chir-OBP3 | XP_005701296.2 | 21.7 | 3 | 71 | Chir-OBP5 | XP_017899538.1 | 9.2 | 2 |
|  | Chir-OBP4 | XP_017899515.1 | 26.9 | 6 |  | Chir-SAL1/2 | XP_0170809(8/9).1 | 16.8 | 3 |
|  | Chir-OBP5 | XP_017899538.1 | 5.3 | 2 |  | Chir-VEG2 | XP_017911671.1 | 23.5 | 4 |
|  | Chir-SAL1/2 | XP_0170809(8/9).1 | 21.6 | 3 | 72 | Chir-OBP6 | XP_017900101.1 | 9.2 | 2 |
|  | Chir-VEG2 | XP_017911671.1 | 38.3 | 4 |  | Chir-SAL1/2 | XP_0170809(8/9).1 | 18.6 | 2 |
| 54 | Chir-OBP2 | XP_017899208.1 | 48.1 | 11 | 73 | Chir-OBP2 | XP_017899208.1 | 30.4 | 3 |
|  | Chir-OBP3 | XP_005701296.2 | 10.2 | 2 |  | Chir-OBP4 | XP_017899515.1 | 19.9 | 2 |
|  | Chir-OBP4 | XP_017899515.1 | 18.6 | 4 |  | Chir-VEG2 | XP_017911671.1 | 23.5 | 2 |
|  | Chir-OBP5 | XP_017899538.1 | 5.3 | 2 | 74 | Chir-OBP2 | XP_017899208.1 | 47.5 | 5 |
|  | Chir-SAL1/2 | XP_0170809(8/9).1 | 15 | 2 |  | Chir-OBP6 | XP_017900101.1 | 18.4 | 2 |
|  | Chir-VEG1 | XP_005687416.1 | 21 | 2 |  | Chir-SAL1/2 | XP_0170809(8/9).1 | 15 | 2 |
|  | Chir-VEG2 | XP_017911671.1 | 34 | 3 |  | Chir-VEG3 | XP_017910286.1 | 15 | 2 |
| 55 | Chir-OBP2 | XP_017899208.1 | 48.1 | 11 | 75 | Chir-OBP2 | XP_017899208.1 | 11.4 | 2 |
|  | Chir-OBP4 | XP_017899515.1 | 13.5 | 3 |  | Chir-OBP3 | XP_005701296.2 | 18.5 | 2 |
|  | Chir-OBP5 | XP_017899538.1 | 5.3 | 2 | 76 | Chir-OBP2 | XP_017899208.1 | 25.3 | 5 |
|  | Chir-SAL1 | XP_017908099.1 | 25.8 | 5 |  | Chir-OBP3 | XP_005701296.2 | 23.6 | 3 |
|  | Chir-SAL2 | XP_017908098.1 | 34.1 | 6 |  | Chir-OBP4 | XP_017899515.1 | 16 | 2 |
|  | Chir-VEG1 | XP_005687416.1 | 21 | 2 |  | Chir-OBP5 | XP_017899538.1 | 20.4 | 3 |
|  | Chir-VEG2 | XP_017911671.1 | 25.9 | 3 |  | Chir-OBP6 | XP_017900101.1 | 18.4 | 2 |
| 56 | Chir-OBP2 | XP_017899208.1 | 43.7 | 8 | 77 | Chir-OBP2 | XP_017899208.1 | 25.3 | 5 |
|  | Chir-OBP4 | XP_017899515.1 | 19.9 | 3 |  | Chir-OBP6 | XP_017900101.1 | 15.8 | 2 |
|  | Chir-OBP5 | XP_017899538.1 | 9.2 | 2 |  | Chir-SAL1/2 | XP_0170809(8/9).1 | 15 | 2 |
|  | Chir-SAL1/2 | XP_0170809(8/9).1 | 17.4 | 2 |  | Chir-VEG3 | XP_017910286.1 | 15 | 2 |
|  | Chir-VEG2 | XP_017911671.1 | 23.5 | 2 | 78 | Chir-OBP2 | XP_017899208.1 | 25.3 | 5 |
| 57 | Chir-OBP2 | XP_017899208.1 | 48.1 | 8 |  | Chir-OBP4 | XP_017899515.1 | 12.2 | 2 |
|  | Chir-OBP3 | XP_005701296.2 | 23.4 | 3 |  | Chir-OBP5 | XP_017899538.1 | 15.8 | 2 |
|  | Chir-OBP4 | XP_017899515.1 | 19.9 | 2 |  | Chir-OBP6 | XP_017900101.1 | 18.4 | 2 |
|  | Chir-SAL1/2 | XP_0170809(8/9).1 | 10.8 | 2 |  | Chir-SAL1/2 | XP_0170809(8/9).1 | 20.4 | 2 |
|  | Chir-VEG2 | XP_017911671.1 | 30.3 | 3 |  | Chir-VEG2 | XP_017911671.1 | 21.6 | 2 |
| 58 | Chir-OBP2 | XP_017899208.1 | 30.4 | 3 | 79 | Chir-OBP2 | XP_017899208.1 | 11.4 | 2 |
|  | Chir-OBP4 | XP_017899515.1 | 19.9 | 2 |  | Chir-OBP5 | XP_017899538.1 | 8.6 | 2 |
|  | Chir-VEG2 | XP_017911671.1 | 23.5 | 2 | 80 | Chir-OBP2 | XP_017899208.1 | 36.1 | 5 |
| 59 | Chir-OBP2 | XP_017899208.1 | 41.1 | 9 |  | Chir-OBP3 | XP_005701296.2 | 10.8 | 2 |
|  | Chir-OBP3 | XP_005701296.2 | 23.6 | 2 |  | Chir-SAL1/2 | XP_0170809(8/9).1 | 15 | 2 |
|  | Chir-OBP4 | XP_017899515.1 | 19.9 | 2 |  | Chir-VEG2 | XP_017911671.1 | 32.7 | 4 |
|  | Chir-OBP5 | XP_017899538.1 | 9.9 | 2 | 81 | Chir-OBP2 | XP_017899208.1 | 38 | 7 |
|  | Chir-SAL1/2 | XP_0170809(8/9).1 | 10.8 | 2 |  | Chir-SAL1/2 | XP_0170809(8/9).1 | 15 | 2 |
|  | Chir-VEG2 | XP_017911671.1 | 30.3 | 3 |  | Chir-VEG2 | XP_017911671.1 | 21.6 | 2 |
| 60 | Chir-OBP2 | XP_017899208.1 | 50 | 10 | 82 | Chir-OBP2 | XP_017899208.1 | 33.5 | 5 |
|  | Chir-OBP4 | XP_017899515.1 | 28.2 | 5 |  | Chir-SAL1/2 | XP_0170809(8/9).1 | 15 | 2 |
|  | Chir-OBP5 | XP_017899538.1 | 9.2 | 2 |  | Chir-VEG2 | XP_017911671.1 | 21.6 | 2 |
|  | Chir-SAL1/2 | XP_0170809(8/9).1 | 21.6 | 3 |  |  |  |  |  |
|  | Chir-VEG2 | XP_017911671.1 | 31.5 | 4 |  |  |  |  |  |

**Table S6** Odorant-binding proteins identified in goat 30432 olfactory secretome in SR (spots 83 to 107, corresponding to Supplementary Fig. S3c) and SA (spots 108 to 145, corresponding to Supplementary Fig. S3d) by nano-LC-MS/MS.

| Spot N° | Protein identification | UniProt KB accession number | % coverage (Peptides matched) | Number of spectra | Spot N° | Protein identification | UniProt KB accession number | % coverage (Peptides matched) | Number of spectra |
| --- | --- | --- | --- | --- | --- | --- | --- | --- | --- |
| 83 | Chir-OBP2 | XP_017899208.1 | 50 (8) | 45 | 110 | Chir-OBP4 | XP_017899515.1 | 19.2 (2) | 2 |
|  | Chir-OBP4 | XP_017899515.1 | 30.8 (6) | 24 | 111 | Chir-OBP4 | XP_017899515.1 | 30.8 (4) | 7 |
| 84 | Chir-OBP2 | XP_017899208.1 | 34.2 (5) | 19 | 112 | Chir-OBP4 | XP_017899515.1 | 25.7 (3) | 7 |
|  | Chir-OBP4 | XP_017899515.1 | 24.4 (4) | 16 | 113 | Chir-OBP4 | XP_017899515.1 | 19.2 (3) | 8 |
| 85 | Chir-OBP1 | XP_017899536.1 | 55.8 (5) | 8 | 114 | Chir-OBP4 | XP_017899515.1 | 19.2 (2) | 2 |
|  | Chir-OBP2 | XP_017899208.1 | 34.2 (6) | 32 | 115 | * |  |  |  |
|  | Chir-OBP4 | XP_017899515.1 | 24.4 (4) | 25 | 116 | * |  |  |  |
| 86 | Chir-OBP2 | XP_017899208.1 | 34.2 (5) | 27 | 117 | Chir-OBP4 | XP_017899515.1 | 19.2 (2) | 3 |
|  | Chir-OBP4 | XP_017899515.1 | 19.2 (3) | 9 | 118 | Chir-OBP4 | XP_017899515.1 | 19.2 (2) | 2 |
|  | Chir-OBP5 | XP_017899538.1 | 14.5 (2) | 2 | 119 | Chir-OBP4 | XP_017899515.1 | 19.2 (3) | 5 |
|  | Chir-OBP6 | XP_017900101.1 | 12.4 (2) | 5 | 120 | * |  |  |  |
| 87 | Chir-OBP2 | XP_017899208.1 | 34.2 (4) | 18 | 121 | Chir-OBP2 | XP_017899208.1 | 19 (2) | 2 |
|  | Chir-OBP4 | XP_017899515.1 | 24.4 (4) | 10 |  | Chir-OBP5 | XP_017899538.1 | 25 (2) | 5 |
|  | Chir-SAL1 | XP_017908099.1 | 15.6 (2) | 2 | 122 | Chir-OBP2 | XP_017899208.1 | 26 (4) | 6 |
| 88 | Chir-OBP2 | XP_017899208.1 | 34.2 (5) | 15 |  | Chir-OBP4 | XP_017899515.1 | 19.2 (2) | 3 |
|  | Chir-OBP4 | XP_017899515.1 | 24.4 (4) | 16 |  | Chir-OBP5 | XP_017899538.1 | 25 (2) | 4 |
| 89 | Chir-OBP2 | XP_017899208.1 | 34.2 (5) | 6 | 123 | Chir-OBP4 | XP_017899515.1 | 19.2 (2) | 2 |
|  | Chir-OBP4 | XP_017899515.1 | 19.2 (3) | 12 | 124 | Chir-OBP1 | XP_017899536.1 | 49.4 (4) | 7 |
| 90 | Chir-OBP2 | XP_017899208.1 | 34.2 (4) | 14 |  | Chir-OBP2 | XP_017899208.1 | 34.2 (5) | 8 |
|  | Chir-OBP4 | XP_017899515.1 | 24.4 (5) | 17 |  | Chir-OBP4 | XP_017899515.1 | 19.2 (3) | 4 |
|  | Chir-SAL1 | XP_017908099.1 | 15.6 (2) | 5 | 125 | * |  |  |  |
| 91 | Chir-OBP2 | XP_017899208.1 | 43.1 (8) | 47 | 126 | Chir-OBP2 | XP_017899208.1 | 51.3 (5) | 49 |
|  | Chir-OBP4 | XP_017899515.1 | 48.7 (8) | 75 | 127 | Chir-OBP2 | XP_017899208.1 | 59.5 (7) | 22 |
| 92 | Chir-OBP2 | XP_017899208.1 | 52.5 (10) | 89 |  | Chir-OBP3 | XP_005701296.2 | 19.1 (2) | 2 |
|  | Chir-OBP4 | XP_017899515.1 | 42.3 (8) | 75 |  | Chir-OBP4 | XP_017899515.1 | 32.1 (5) | 25 |
| 93 | Chir-OBP2 | XP_017899208.1 | 52.5 (9) | 135 | 128 | Chir-OBP2 | XP_017899208.1 | 43.1 (7) | 50 |
|  | Chir-OBP4 | XP_017899515.1 | 30.8 (6) | 41 |  | Chir-OBP4 | XP_017899515.1 | 25.7 (4) | 30 |
| 94 | Chir-OBP2 | XP_017899208.1 | 79.1 (11) | 123 | 129 | Chir-OBP1 | XP_017899536.1 | 20.5 (2) | 2 |
|  | Chir-OBP4 | XP_017899515.1 | 30.8 (5) | 32 |  | Chir-OBP2 | XP_017899208.1 | 43.1 (7) | 56 |
| 95 | Chir-OBP2 | XP_017899208.1 | 42.4 (5) | 122 |  | Chir-OBP4 | XP_017899515.1 | 30.8 (4) | 30 |
|  | Chir-OBP4 | XP_017899515.1 | 25.7 (3) | 7 | 130 | Chir-OBP2 | XP_017899208.1 | 43.1 (7) | 39 |
| 96 | Chir-OBP2 | XP_017899208.1 | 26.5 (4) | 77 |  | Chir-OBP4 | XP_017899515.1 | 30.1 (5) | 28 |
| 97 | Chir-OBP2 | XP_017899208.1 | 43.1 (8) | 60 | 131 | Chir-OBP2 | XP_017899208.1 | 68.4 (8) | 30 |
|  | Chir-OBP4 | XP_017899515.1 | 37.2 (9) | 41 |  | Chir-OBP4 | XP_017899515.1 | 30.1 (5) | 10 |
|  | Chir-VEG2 | XP_017911671.1 | 48 (4) | 15 | 132 | Chir-OBP2 | XP_017899208.1 | 68.4 (8) | 25 |
| 98 | Chir-OBP2 | XP_017899208.1 | 52.5 (10) | 83 |  | Chir-OBP4 | XP_017899515.1 | 19.2 (2) | 5 |
|  | Chir-OBP4 | XP_017899515.1 | 37.2 (8) | 39 | 133 | Chir-OBP2 | XP_017899208.1 | 43.1 (7) | 26 |
|  | Chir-VEG2 | XP_017911671.1 | 40.2 (4) | 8 |  | Chir-OBP4 | XP_017899515.1 | 25.7 (3) | 10 |
| 99 | Chir-OBP2 | XP_017899208.1 | 53.2 (12) | 135 | 134 | Chir-OBP2 | XP_017899208.1 | 43.1 (7) | 28 |
|  | Chir-OBP4 | XP_017899515.1 | 42.3 (7) | 45 |  | Chir-OBP4 | XP_017899515.1 | 25.7 (4) | 10 |
|  | Chir-VEG2 | XP_017911671.1 | 29.9 (3) | 7 |  | Chir-VEG2 | XP_017911671.1 | 37.8 (3) | 6 |
| 100 | Chir-OBP2 | XP_017899208.1 | 50 (8) | 63 | 135 | * |  |  |  |
|  | Chir-OBP4 | XP_017899515.1 | 30.8 (6) | 31 | 136 | Chir-OBP2 | XP_017899208.1 | 34.2 (5) | 13 |
|  | Chir-VEG2 | XP_017911671.1 | 37.8 (3) | 5 |  | Chir-OBP4 | XP_017899515.1 | 19.2 (3) | 4 |
| 101 | Chir-OBP2 | XP_017899208.1 | 28.5 (5) | 38 | 137 | Chir-OBP2 | XP_017899208.1 | 34.2 (6) | 15 |
|  | Chir-OBP4 | XP_017899515.1 | 19.2 (3) | 6 |  | Chir-OBP4 | XP_017899515.1 | 19.2 (3) | 8 |
| 102 | Chir-OBP2 | XP_017899208.1 | 36.1 (6) | 40 | 138 | Chir-OBP4 | XP_017899515.1 | 24.4 (4) | 19 |
|  | Chir-OBP4 | XP_017899515.1 | 19.2 (3) | 10 | 139 | Chir-OBP2 | XP_017899208.1 | 19.6 (2) | 3 |
| 103 | Chir-OBP2 | XP_017899208.1 | 43.6 (8) | 35 | 140 | Chir-OBP2 | XP_017899208.1 | 19.6 (3) | 5 |
|  | Chir-OBP4 | XP_017899515.1 | 19.2 (3) | 7 |  | Chir-OBP4 | XP_017899515.1 | 19.2 (2) | 2 |
| 104 | Chir-OBP2 | XP_017899208.1 | 36.1 (6) | 42 | 141 | * |  |  |  |
|  | Chir-OBP4 | XP_017899515.1 | 19.2 (2) | 4 | 142 | Chir-OBP2 | XP_017899208.1 | 19.6 (3) | 6 |
| 105 | Chir-OBP2 | XP_017899208.1 | 22.8 (4) | 23 |  | Chir-OBP4 | XP_017899515.1 | 19.2 (2) | 2 |
| 106 | * |  |  |  | 143 | Chir-OBP2 | XP_017899208.1 | 15.2 (2) | 2 |
| 107 | * |  |  |  | 144 | Chir-OBP1 | XP_017899536.1 | 64.1 (7) | 10 |
| 108 | Chir-OBP2 | XP_017899208.1 | 34.2 (6) | 13 |  | Chir-OBP2 | XP_017899208.1 | 34.2 (6) | 12 |
|  | Chir-OBP4 | XP_017899515.1 | 19.2 (2) | 5 |  | Chir-OBP4 | XP_017899515.1 | 19.2 (2) | 5 |
| 109 | Chir-OBP2 | XP_017899208.1 | 43.1 (6) | 17 | 145 | Chir-OBP2 | XP_017899208.1 | 34.2 (5) | 7 |
|  | Chir-OBP4 | XP_017899515.1 | 25.7 (4) | 11 |  | Chir-OBP4 | XP_017899515.1 | 19.2 (2) | 7 |

**Table S7** Monitoring of progesterone concentration in blood of the ewes and goats used in this study

| Animals | March 2016  (SR) | | | | November 2016  (SA) | | | | March 2017  (SR) | | | | November 2017 (SA) | | | March 2018  (SR) | | | | November 2018  (SA) | | | |
| --- | --- | --- | --- | --- | --- | --- | --- | --- | --- | --- | --- | --- | --- | --- | --- | --- | --- | --- | --- | --- | --- | --- | --- |
|  | Progesterone  (ng/ mL) | | | status | Progesterone  (ng/ mL) | | | status | Progesterone  (ng/ mL) | | | status | Progesterone  (ng/ mL) | | status | Progesterone  (ng/ mL) | | | status | Progesterone  (ng/ mL) | | | status |
|  | D1 | D7 | D13 |  | D1 | D7 | D13 |  | D1 | D2 | D7 |  | D1 | D7 |  | D1 | D7 | D11 |  | D1 | D7 | D13 |  |
| Ewe 30056 | <0.25 | <0.25 | 0.8 | A | 0.7 | 4.0 | 5.1 | O | 1.4 | 3.1 | 4.8 | O | 2.0 | 3.9 | O |  |  | <0.25 | A | 3.4 | 2.8 | 0.4 | O |
| Ewe 30094 | <0.25 | <0.25 | <0.25 | A | 2.1 | 1.1 | 2.7 | O | <0.25 | <0.25 | <0.25 | A | <0.25 | 2.3 | O |  |  | <0.25 | A | 3.2 | <0.25 | 2.1 | O |
| Ewe 30118 | <0.25 | <0.25 | <0.25 | A | 4.5 | <0.25 | 2.6 | O | <0.25 | <0.25 | <0.25 | A | 3.8 | <0.25 | O |  |  | <0.25 | A | 2.9 | 0.3 | 0.9 | O |
| Goat 30422 |  |  |  |  | <0.25 | 4.5 | 6.4 | O | <0.25 | <0.25 | <0.25 | A | 12.1 | 9.7 | O | 0.8 | 9.2 | 7.5 | O | 0.3 | 9.8 | 8.0 | O |
| Goat 30432 |  |  |  |  | 1.7 | 1.5 | 9.6 | O | <0.25 | <0.25 | <0.25 | A | 0.4 | 3.9 | O | 1.3 | <0.25 | 5.8 | O | <0.25 | 6.2 | 7.5 | O |
| Goat 30363 | <0.25 | <0.25 | 0.3 | A | 0.4 | <0.25 | <0.25 | A | <0.25 | <0.25 | 1.0 | A | 2.5 | 13.3 | O | <0.25 | <0.25 | <0.25 | A | 12.1 | 9.3 | 8.8 | O |

D = day of blood sampling; status means physiological status: O = oestrus, A = anoestrus.

**Table S8** Primers used for amplification of the major OBPs expressed in ewe and goat olfactory secretome

|  | | 5’ primer (forward) | 3’ primer (reverse) |
| --- | --- | --- | --- |
| Oari-OBP2 | 3’ RACE PCR | Gene Specific 3’ primer  5’-GGGCAAAGATGAAGTTGACGACCATGAT-3’ | GeneRacer™ 3’ primer  5’-GCTGTCAACGATACGCTACGTAACG-3’ |
|  | Full-length  amplification | 5OariOBP2  5’-ATGAAGGTCCTGTTTTTGACGTTGGTCCTTGGT-3’ | 3OariOBP2  5’-TCACTGGGCCGACAGCCGTCACCCTT-3’ |
| Oari-OBP4 | 3’ RACE PCR | Gene Specific 3’ primer  5’-AGATCACAAAATTAACTGAAGGTTGCGCCAAAGG-3’ | GeneRacer™ 3’ primer  5’-GCTGTCAACGATACGCTACGTAACG-3’ |
|  | 5’ RACE PCR | GeneRacer™ 5’ primer  5’-CGACTGGAGCACGAGGACACTGA-3’ | Gene Specific 5’ primer  5’-GATTGTAACACCTCAGCGGGCCCCCTT-3’ |
|  | Full-length  amplification | 5OariOBP4  5’-ATGAAGGTTCTGTTGCTGAGTCTCGTC-3’ | 5ChirOBP4  5’-TTATGGAGGACAGTCGTCTGTTTCGATGAC-3’ |
| Chir-OBP2 | 3’ RACE PCR | Gene Specific 3’ primer  5’-AACAGAACCAGACTCATTGGGCTCTTGGGCAA-3’ | GeneRacer™ 3’ primer  5’-GCTGTCAACGATACGCTACGTAACG-3’ |
|  | Full-length amplification | 5ChirOBP2  5’-ATGCAAGCCAACAAGATGAAGGTCCTGTTTTT-3’ | 3ChirOBP2  5’-TCACTGGGCCGACAGCCGTCACCCTT-3’ |
| Chir-OBP4 | 3’ RACE PCR | Gene Specific 3’ primer  5’-GATCACAAAATTAACTGAAGGTTGCGCCAAAGGA-3’ | GeneRacer™ 3’ primer  5’-GCTGTCAACGATACGCTACGTAACG-3’ |
|  | Full-length amplification | 5ChirOBP4  5’-ATGAAGGTTCTGTTGCTGAGTCTCGTC-3’ | 3ChirOBP4  5’-TTATGGAGGACAGTCGTCTGTTTCGATGAC-3’ |

**Figure S1** Sequence alignment of predicted lipocalins from sheep and goat genomes (BlastX searches). Alignments have been performed with Multalin (<http://multalin.toulouse.inra.fr/multalin>; (22)). Sequences are divided into 3 groups, OBP, SAL, and VEG (accession numbers in the text). Cysteines are underlined in yellow, GxW motif in blue, and additional GxW motif of bovine OBP-like sequences in green.

1. **OBP group**

1 100

W5PH68 QEKEVEQNPS ELSGQWRTVY IGSTDPEKIQ EDGPFRTYFH KIVFDDEK.G TVDFYFYVKQ NGKWKNVHVT GTKQDDGTYS VEYEGQNEFK VLSVSKTHLV

P07435-OBP-BOVIN QEEEAEQNLS ELSGSWRTVY IGSTNPEKIQ ENGPFRTYFR ELVFDDEK.G TVDFYFSVKR DGKWKNVHVK ATKQDDGTYV ADYEGQNVFK IVSLSRTHLV

W5PGV5 QEEEAQPSLS ELSGQWRTAY IASSNLGKIK PNGPFRVYLQ KLLFDDEQ.G TIDFYFYVKH KGKWEYKHVT GIKQDDGTFA VDYEGENVFA VTHASRNILV

W5PZN0 QEADDQKTLS QLSGKWRTVY IASTRPEKIA EDGPFRAHVR YLVFDGEQ.G TVDFYFYVKL NGEWVAKHVT GQKTENNTYV VEYEGENKFE VIYASDTVLV

W5PHA2 ......QDEA QFSGEWRTHY IASSNIEKIT ENGPFHIYAR YIQFNADN.. TVDVDFYIKS NGECIKKHET AQKQQNFTYT AEYAGHNEGR VLHVSHNSVI

XP_017899539.1 .....QIDAS QFTGRWLTYY MAANNIEKIT EGAPFYAFMR YIEFDEEN.G TILMHFYVKE NGECIEKYVS GTKEGNF.YA VDYAGHNEFQ LIRGDENTLL

XP_017899538.1 .....QIDAS QFTGRWLTHY IAADNVEKIT EGAPFHIFMR YIEFDEEN.G TVHFHFYIKK NGECIEKYVS GLKEEAH.YA IDYAGHNEFQ LISGDKDYLL

XP_017900101.1 .....QIDAS QFTGRWLTHY IAADNVEKIT EGVPFHIFMR YIEFDEEN.G TVHFHFYIKK NGQCIEKYTS GLKEETH.YA IDYAGHNEFQ LISGDKDYLI

W5PGN0 QEIPAEPHHS EISGEWRTHY IASSNTEKTG ENGPFNVYLR SIKFNERGLP CLPLLCQVRN NGECTESSVS GRRIANNVYV AEYAGANEFH FILVSDDGLI

XP_017899208.1 QEIPAEPHHS QISGEWRTHY IASSNTDKTG ENGPFNVYLR SIKFNDKG.D SLVFHFFVKN NGECTESSVS GRRIANNVYV AEYAGANQFH FILVSDDGLI

P81245-OBP-PIG QEPQPEQDPF ELSGKWITSY IGSSDLEKIG ENAPFQVFMR SIEFDDKE.S KVYLNFFSKE NGICEEFSLI GTKQEGNTYD VNYAGNNKFV VSYASETALI

W5PHS2 QETPAEIDPS KVTGEWRTIY SAADNKEKIV EGGPLRCYNR KIECTDDC.E HLSISFYVKF DGRCQFFSGV LKRQEGGVYF IEFEGANYLQ IIHVSDNILV

XP_017899515.1 QETPAEIDPS KVTGEWRTIY SAADNKEKIV EGGPLRCYNR KIECIDDC.E YLSISFYVKL DGRCQFFSGV LKRQEGGVYF IEFAGANYLQ IIHVSDNILV

XP_017899516.1 QETPAEIDPS KVTGEWRTIY SAADNKEKIV EGGPLRCYNR KIECIDDC.E YLSISFYVKL DGRCQFFSGV LKRQEGGVYF IEFAGANYLQ IIHVSDNILV

WPPHN1 QETPAEIDPS KIPGEWRTIY TAADNKDKIV EGGPLRNYYR RIECIDDC.E SLSITFYLKD DGTCLLLTEV AKRKEGYVYV IEFDGTNTLE VIHVSENMLV

XP_005701296.1 QESPAEIDPS KIPGEWRTIY AAADNKDKIV EGGPLRNYYR RIECINDC.E SLSITFYLKD DGTCLLLTEV AKGTEGYVYV IQFDGTNTLE VIHVSENMLV

XP_017899207.1 PETATEIDPS KVTGEWHTIY AAADNKEKIV KGGLLRCYYH QIECINDC.K YPSLTFYAKD DGRCQLFTEV RKRQEGDVYV IEFMGTNVLQ LIYVSDNMVV

W5PHM2 QEAPAELDPS QITGDWRSIL TAADNKEKIE EEGPLRTYVR RLECIDSC.S SLSIKFYAKF PKQCTFLNIV AER.EGDVYQ VGYMGSNSFK LILVSENSLA

XP_017899536.1 QEAPAQPESS EITAKWYTIY MAADHKEKIE EGGPLRTYFR QLECIDSC.E KMSITFILTN YDSCTLITVV AQRAEENVYH VDYMGKNSVQ LIPASESMLV

W5PGW3 .HGEEEGGSS RSFTGINSLN SAAGRGSCPG TNQGLEPLLQ S..FLETGKA SAIFTIFVSS NGECVKKQVT GEKEKISVYH ITYAGQNKVK ILRLSLDTII

Consensus qe...#.d.s ...g.Wrt.y iaa.n.eKi. e.gpfr.y.r .ief.d.... .....fyvk. ng.c....v. g.k.e..vY. ..%aG.N.f. .i..s...l.

111 178

W5PH68 AHNFNVDKQG K..ETELTGL FVKFN.VEDE DEDLEKFRTE DQGSDRKYVV TFAENEDHLH SE........ ........

P07435-OBP-BOVIN AHNINVDKHG Q..KTELTGL FVKLN.VEDE DLEKFWKLTE DKGIDKKNVV NFLENEDHPH PE........ ........

W5PGV5 AHNINVDEHG K..KTVLTGL FVKVN.IEEE GLQKFKELTQ EKGIKEKNVV NFIETDD... .......... ........

W5PZN0 VSVVNKDKNK KCGEIQLAGI FVKVNDIEEK ALEIFKELLK LKGIEEKYIV NFFKGGDCPP CA........ ........

W5PHA2 GHLINVDEEG N..ETDFNWL SGTDDEISDS DFERFKEETR DKGIPEENII NFIDNDDCPE .......... ........

XP_017899539.1 THTVNVDEHG K..ETELVQL FGTGNNVESQ DKEEYYNAVR EKEIPEENIV NFIDTDNCPE E......... ........

XP_017899538.1 VRHLNVDADG K..ETELVGL FGAGNNVDPK HEEEFRNAVR ERGIPEENIQ NFIDNDDCPE E......... ........

XP_017900101.1 ARDLNVDADG K..ETELVGL FGAGNNVDPK HEEEFRNAVR ERGIPEENIR NFIDNDDCPE E......... ........

W5PGN0 VNSENVDEAG N..RTRLVGL LGKEDEVDDH DLERFLEEVR KLGIPEENIV DFTKGDCSLA .......... ........

XP_017899208.1 VNTENVDDEG N..RTRLIGL LGKEDEVDDH DLERFLEEVR KLGIPEENIV DFTKGGRASR VT........ ........

P81245-OBP-PIG ISNINVDEEG D..KTIMTGL LGKGTDIEDQ DLEKFKEVTR ENGIPEENIV NIIERDDCPA K......... ........

W5PHS2 LYFENDDGQK I...TKLTEG CAKGTSFTQE EFQKYQQLNS EREVPNENIE HVIETDDCPP .......... ........

XP_017899515.1 LYFENDDGEK I...TKLTEG CAKGTSFTQE EFQKYQQLNS ERGIPNENVE HVIETDDCPP .......... ........

XP_017899516.1 LYFENDDGEK I...TKLTEG CAKGTSFTQE EFQKYQQLNS ERGIPNENVE HVIETDDCPP .......... ........

WPPHN1 TYAENYDGER I...TKMTEG LAKGTSFTQE ELQKYQQLNS EREVPNENIE NVIETGKAPE DDRFP..... ........

XP_005701296.1 TYVENYDGER I...TKMTEG LAKGTSFTQE ELQKYQQLNS ERGVPNENIE NVIETGKAPE DDRFPK.... ........

XP_017899207.1 TYFENDDGEK I...TKITEG VGRGDSFTQE ELQKYPELNS KRGIPNENIE NVIKTGSKKK MKGEHEQHRS RNVSQSYV

W5PHM2 VYGENFDGVK V...TKVTQL LAKGDGTTEE ETQQYEELNK ERGIPPEHVK DLTQTDNCPQ .......... ........

XP_017899536.1 FYAENFDGEK T...TKVTYA LGKGDSLSQE DIQKYEEINN ERGIPNENTE DGSNTDNCPK .......... ........

W5PGW3 GSIHNVDEDG K..ETELVGI LGKRDQISDI DYEKFKKEAS DRGIPEENIV NFTDNDDCPA E......... ........

Consensus ....NvD..g ....T.ltgl .gkg...... d.#k%.e... e.g!peen!v nfi..ddcp. .......... ........

1. **SAL**

1 87

W5P8Y1 .......... ....QEGTSD VVRSNFDIPK IAGEWYSILL ASDHREKIE. ENGSMRVFVE HIDVLENSSL SFKFHTKVNG VCTELPLVSD STGEDGVYTI

XP_017908099.1 .......... ....QEGTSD VVRSNFDIPK IAGEWYSILL ASDHREKIE. ENGSMRVFVE HIDVLENSSL SFKFHTKVNG VCTELPLVSD STGEDGVYTI

W5P8W4 .......... ....QEGNSD VVRSNFDIPK IAGEWYSILL ASDNREKIE. ENGSMRFFVE HISLLENSSL FIKMHTKVNG VCTELPLTCD STGEDGVYTV

XP_017908098. .......... ....QEGNSD VVRSNFDIPK ITGEWFSILL ASDNREKIE. ENGSMRFFVE HISLLENSSL FIKMHTKVNG VCTELPLTCD STGEDGVYTV

P81608-SAL-PIG .......... ...HKEAGQD VVTSNFDASK IAGEWYSILL ASDAKENIE. ENGSMRVFVE HIRVLDNSSL AFKFQRKVNG ECTDFYAVCD KVG.DGVYTV

W5P4T6 .......... ...QDFNPQR IVQRNYDVSK VSGTWYSISM AADNRKRIE. EDGDLRIFIE SIQVVEDGRL KLSFHFMLHA ECTDVAMVCG KTGKNGEYTI

AHZ46504.1 .......... ...QDFNPRR IVQRNYDVSK VSGTWYSISM AADNRKRIE. EDGDLRIFIE SIQVIEDSGL KLSFHFMLHA ECTDVAMVCG KTGKNGEYTI

XP_017910280.1 .......... ...QDFNPRR IVQRNYDVSK VSGTWYSISM AADNRKRIE. EDGDLRIFIE SIQVVEDSGL KLSFHFMLHA ECTDVAMVCG KTGKNGEYTI

W5P4W8 QACCLSPPLP AELRSTTPAA ETMKGLDIQK VAGTWHSLAM AASDISLLDA QSAPLRVYVE ELKPTPEGNL EILLQKWENG ECAQKKIIAE KTKIPAVFKI

Consensus .......... .......p.. .v..n.Di.K !aGtWySi.$ Aad.r..i#. #.g.$Rv%!E .i.v.e#s.L ...fh...ng eCt#...vc. ktg..gv%t!

88 180

W5P8Y1 SYDGNNTFRI LQVNYSRHII FYLENFSD.. SYQLLELYAR EPDTSPELKN EFVEICQKYG IVKENIIDLT RVDRCFQARG NGVA...... ...

XP_017908099.1 SYDGNNKFRI LQVNYSHHII FYLENFSD.. SYQLLELYAR EPDTSPELKN EFVEICQKYG IVKENVIDLT KVDRCFQARG NGVA...... ...

W5P8W4 SYDGNNKFRI LQVNYSHHII FYLENFSD.. SFQLLELYAR EPDTSPELKN EFVEICQKYG IVKENVIDLT RVDRCFQARG NGVA...... ...

XP_017908098.1 SYDGNNKFRI LQVNYSHHII FYLENFSD.. SYQLLELYAR EPDTSPELKN EFVEICQKYG IVKENVIDLT KVDRCFQARG NGVA...... ...

P81608-SAL-PIG AYYGENKFRL LEVNYSDYVI LHLVNVNGDK TFQLMEFYGR KPDVEPKLKD KFVEICQQYG IIKENIIDLT KIDRCFQLRG SGGVQESSAE ...

W5P4T6 NYLGENSLRI LEADYQRYVI LHMQSSRNGT ASQVLALYGR FPELKSSFLD RFDKACKSHG LGPEKIIRFS NQDPCY.A.. .......... ...

AHZ46504.1 NYLGENSLRI LEADYQRYVI LHMQSSRNGT ASQVLALYGR FPELKSSFLD RFDKACKSHG LGPEKIIRFS NQDPCY.ARS RYPSRPSVLE PTG

XP_017910280.1 NYLGENSLRI LEADYQRYVI LHMQSSRNGT ASQVLALYGR FPELKSSFLD RFDKACKSHG LGPEKIIRFS NQDPCY.ARS RYPSRPSVLE PTG

W5P4W8 DALNENKVLV LDTDYKKYLL FCMENSAEPE QSLACQCLVR TPEVDNEALE KFDKALKALP PAPQASQEEG QCHV...... .......... ...

Consensus .ylg#Nk.ri L#.#Y..y.i f.$ens.... .sq.l.ly.R .P#...e.l# .Fdkack..g ..p#.ii... ..d.c..ar. .......... ...

1. **VEG**

1 100

W5NUS5 QDALVLDSWT EDVSGKWYLK AVTTDQDVPG KNQESV..TA MTFSVLEGGD LEAKVTLRV. DGQCQETGLV LEQTNDPGRY T.AYGGKREV FILPLRAQDH

XP_005687416.1 QDAPVLDSWT EDVSGKWYLK AVTTDQDVPG KNQELV..TA MTFSVLEGGD LEAKVTLRV. DGQCQETGLV LEQTNDPSRY T.AYGGKHEV FILPLRAQDH

P53715-PIG-VEG QEFPAVGQPL QDLLGRWYLK AMTSDPEIPG KKPESV..TP LILKALEGGD LEAQITFLI. DGQCQDVTLV LKKTNQPFTF T.AYDGKRVV YILPSKVKDH

P20289-VEG-RAT .QAFPTTEEN QDVSGTWYLK AAAWDKEIPD KKFGSVSVTP MKIKTLEGGN LQVKFTVLI. AGRCKEMSTV LEKTDEPAKY T.AYSGKQVL YIIPSSVEDH

P31025-VEG-HUMAN HHLLASDEEI QDVSGTWYLK AMTVDREFPE MNLESV..TP MTLTTLEGGN LEAKVTMLI. SGRCQEVKAV LEKTDEPGKY T.ADGGKHVA YIIRSHVKDH

W5NV32 ..QDNLSFQE PDVLGKWFIT AVVETEGLTG DRV.....FP ITFSALSDTH VWASTTLRT. RGFCYDVDVV LEKTSRSGTY T.ASRGKTHV EVEELPTKDH

XP_017910286.1 ..QDNLSFQG PDVLGKWFIT AVVETEGLTG DRV.....FP ITFSVLSDTH VWASTTLRT. RGFCYDVDVV LEKTSGSGTY T.ASRGKTHV EVEELPAEDH

XP_017911671.1 .......... .......... .......... .....MKMPV VLVTSLANGN LGIKFGFPTP DGGCQETDST FTRGAVDGQF SNAAMAQTDI RVAFTDYKHF

Consensus .......... .dv.g.w... a......... .........p .....L..gn l.ak.t..t. dG.Cq#.d.v lekt...g.% t.A..gkt.. .!.....kdh

101 162

W5NUS5 FILYCEGELG GRQIRVARLL GRNPENSPEA WEEFTEFAKA KKLNLK.IFR PLQSETCSPR GN

XP_005687416.1 FILYCEGELD GRQIRVARLL GRNPENSPEA WEEFTEFAKA KKLNLE.IFR PLQSETCSPR GN

P53715-PIG-VEG YILYCEGELD GQEVRMAKLV GRDPENNPEA LEEFKEVARA KGLNPD.IVR PQQSETCSPG GN

P20289-VEG-RAT YIFYYEGKIH RHHFQIAKLV GRDPEINQEA LEDFQSVVRA GGLNPDNIFI PKQSETCPLG SN

P31025-VEG-HUMAN YIFYCEGELH GKPVRGVKLV GRDPKNNLEA LEDFEKAAGA RGLSTESILI PRQSETCSPG SD

W5NV32 LMFYCEGPFE AGRFRAAKLL SRNPDVNPE. .......... .......... .......... ..

XP_017910286.1 LMFYCEGPFE AGRFRAAKLL SRNPDVNPEA LEAFKKFAQR KGLSPEDIFT PEQTESCKPE SD

XP_017911671.1 AVMYFETQKG AVRNTWLQLY ARAPELFPEG AQRMRELAPK VGLNPSQGVL LPKSDQCAEV LA

Consensus ...YcEg... a.r.r.akL. .R.Pe.npEa .e.f.e.a.. .glnp..i.. p.qse.c... ..

**Figure S2** Two-dimensional electrophoresis of soluble proteins extracted from nasal mucus of ewes 30094 (**a, b**) and 30056 (**c, d**) in sexual rest (SR) and sexual activity (SA) periods. Coomassie blue staining. Molecular weight markers: Precision Plus Protein Standard All blue (Bio-Rad), except for ewe 30056 SR: Precision Plus Protein Standard unstained (Bio-Rad). Protein spots were cut out for protein identification according to the map below (numbering corresponds to Table 1, Table S2 (**a, b**), and Table S3 (**c, d**)).

**
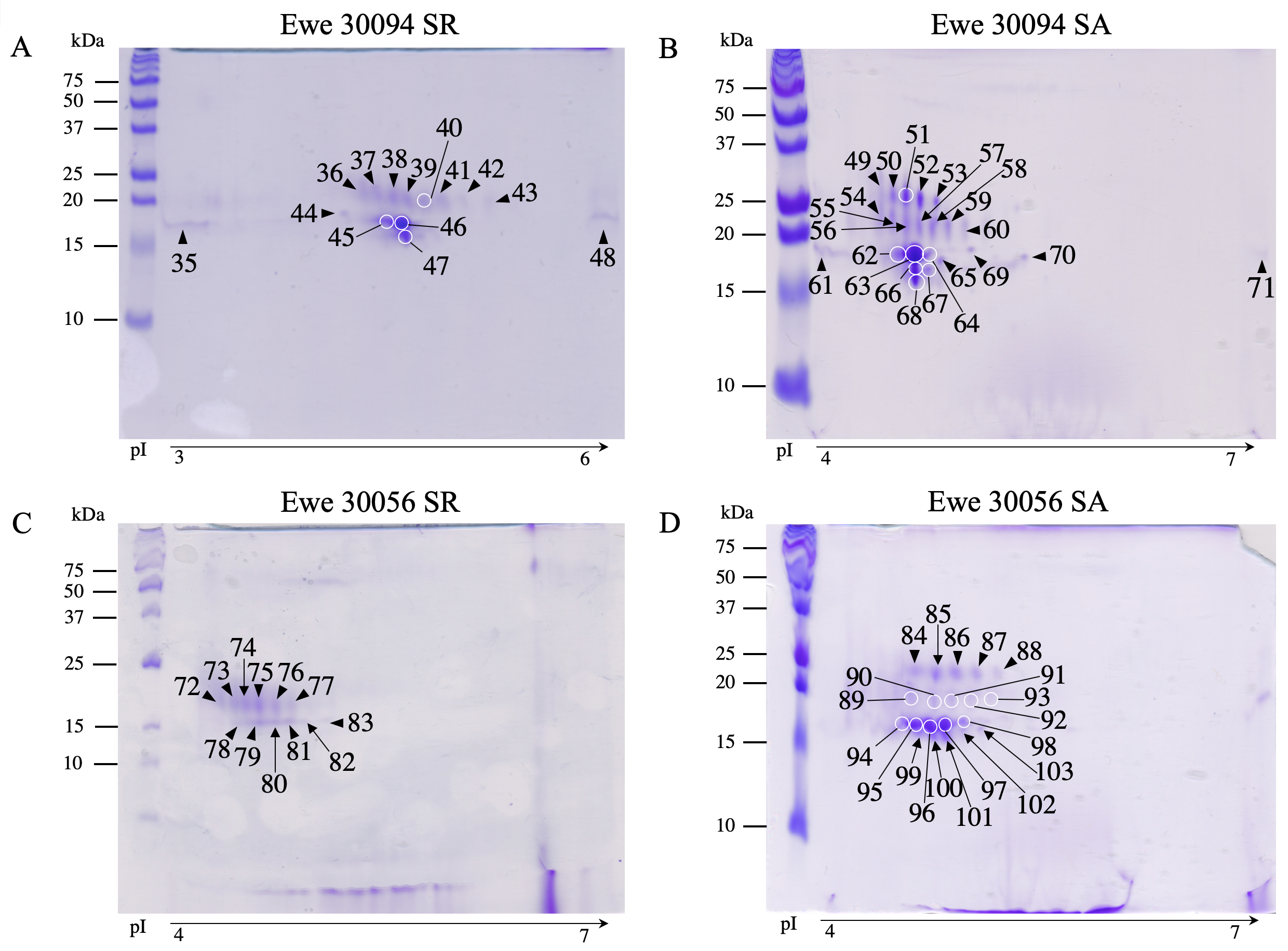
**

**Figure S3** Two-dimensional electrophoresis of soluble proteins extracted from nasal mucus of goats 30422 (**a, b**) and 30432 (**c, d**) in sexual rest (SR) and sexual activity (SA) periods. Coomassie blue staining. Molecular weight markers: Precision Plus Protein Standard All blue for goat 30432 (Bio-Rad), and Precision Plus Protein Standard unstained (Bio-Rad) for goat 30422. Protein spots were cut out for protein identification according to the map below (numbering corresponds to Table 2, Table S5 (**a, b**), and Table S6 (**c, d**)).

**
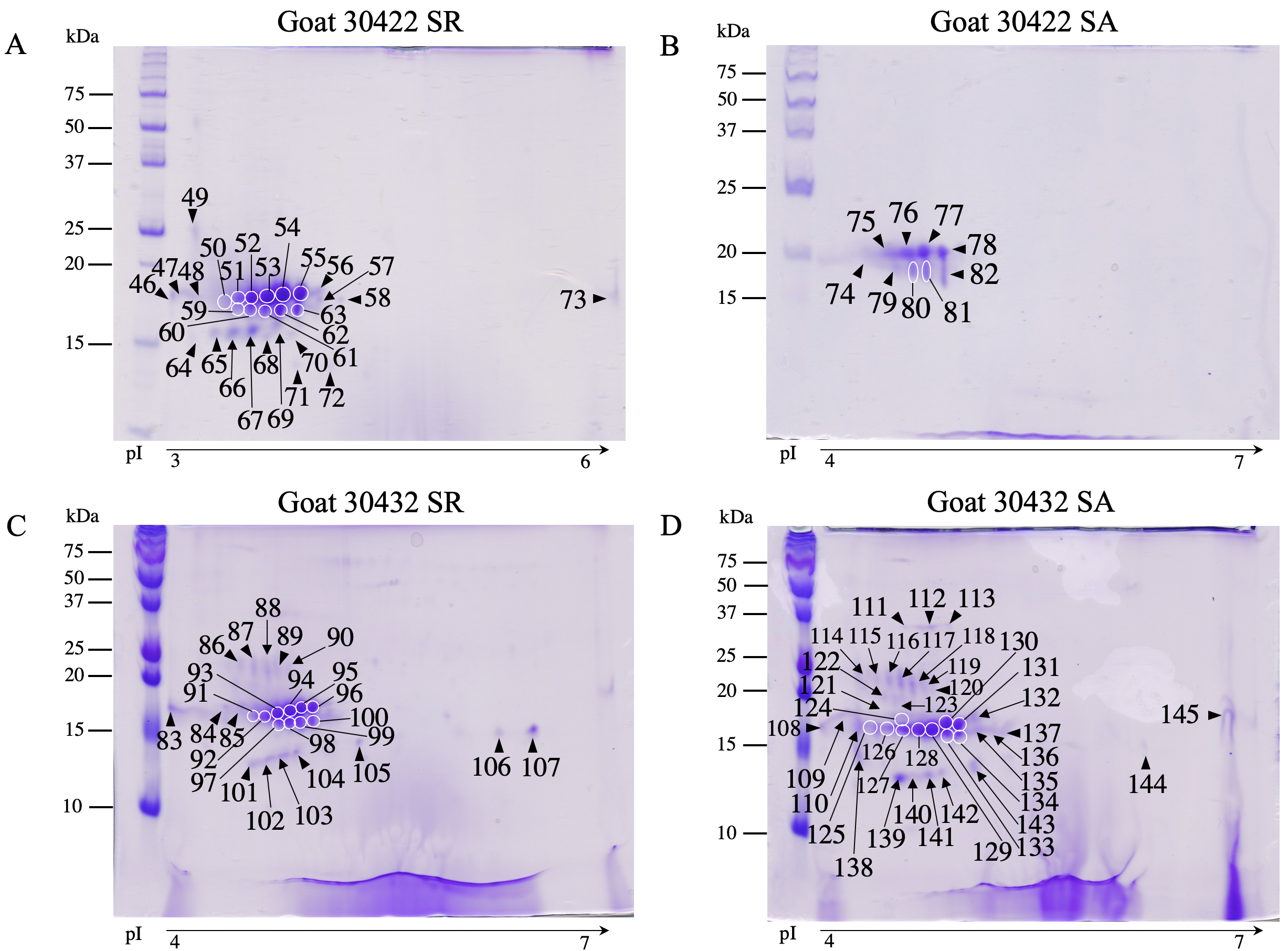
**

**Figure S4** Full-length nucleotide and translated amino acid sequences of Chir-OBP2 and Chir-OBP4 obtained by RACE-PCR. Signal peptide is italicized and cysteines are in bold.

| Chir-OBP2  ATGAAGGTCCTGTTTTTGACTCTGGTCCTTGGTCTGGTCTGTTCTTCCCAGGAG  *M K V L F L T L V L G L V C S S*  Q E  ATTCCAGCTGAGCCACACCACTCACAGATTTCAGGAGAATGGAGAACTCATTAC  I P A E P H H S Q I S G E W R T H Y  ATCGCCTCCAGCAACACAGACAAGACCGGTGAGAACGGGCCATTCAACGTTTAT  I A S S N T D K T G E N G P F N V Y  CTTCGCAGCATCAAATTTAACGACAAAGGGGACTCCCTTGTCTTCCACTTCTTT  L R S I K F N D K G D S L V F H F F  GTCAAGAACAATGGGGAATGTACAGAGTCATCTGTTAGTGGAAGAAGAATCGCA  V K N N G E **C** T E S S V S G R R I A  AACAACGTTTACGTGGCTGAATATGCGGGTGCCAATGAATTCCACTTTATTCTG  N N V Y V A E Y A G A N E F H F I L  GTGTCTGACGATGGTCTGATAGTAAATACTGAAAACGTGGATGATGAAGGCAAC  V S D D G L I V N T E N V D D E G N  AGAACCAGACTCATTGGGCTCTTGGGCAAAGAAGATGAAGTTGACGACCATGAT  R T R L I G L L G K E D E V D D H D  CTGGAGAGGTTCCTTGAGGAGGTTAGAAAATTGGGGATTCCAGAGGAAAATATT  L E R F L E E V R K L G I P E E N I  GTGGATTTCACCAAGGGTGACGGCTGTCAGGCCCAGTGAAAAAAAAAAAAGAGT  V D F T K G D G **C** Q A Q -  GATGTCAACTCCACCTAATTAGTCTGTGAGACCGGACACATCCTCTTCCCCATG  GAATCAAGATCAACAACACGAAGACGATTTCTTCCTGGTTCCCGATCGACATGA  TCCTTCTCTCTTCACACTCACACTCTCTTTCCCATCTCATCTCTCCTGCTCACT  ACTCACTTCCTGTCTGTTGGTGTTTGATTTCTGAGGGTTTAAATAAACTGGTTT  AATACAAAAAAAAAAAAAAAAAAAAAAAAAA | Chir-OBP4  ATGAAGGTTCTGTTGCTGAGTCTCGTCCTTGTCCTGGTTTGTGCCGCCCAGGAA  *M K V L L L S L V L V L V C A A*  Q E  ACTCCAGCTGAGATAGACCCCTCAAAGGTTACAGGCGAGTGGCGCACCATTTAT  T P A E I D P S K V T G E W R T I Y  TCGGCCGCGGATAACAAGGAGAAGATTGTGGAAGGGGGCCCGCTGAGGTGTTAC  S A A D N K E K I V E G G P L R **C** Y  AATCGCAAGATCGAATGTATTGATGACTGCGAATACCTCTCCATTTCATTTTAC  N R K I E **C** I D D **C** E Y L S I S F Y  GTCAAATTAGATGGGAGATGCCAGTTTTTCTCAGGAGTGCTAAAGAGACAAGAA  V K L D G R **C** Q F F S G V L K R Q E  GGAGGTGTTTACTTCATAGAATTTGCAGGTGCAAATTATTTGCAAATCATTCAT  G G V Y F I E F A G A N Y L Q I I H  GTATCAGACAACATCCTGGTACTTTATTTTGAAAATGATGATGGAGAGAAGATC  V S D N I L V L Y F E N D D G E K I  ACAAAATTAACTGAAGGTTGCGCCAAAGGAACCAGTTTCACTCAAGAAGAATTT  T K L T E G **C** A K G T S F T Q E E F  CAGAAGTATCAGCAGCTGAACAGTGAGAGAGGGATTCCAAATGAAAATGTAGAA  Q K Y Q Q L N S E R G I P N E N V E  CATGTCATCGAAACAGACGACTGTCCTCCATAAGAACAGAGAACATCAGCTGGC  H V I E T D D **C** P P -  AGAAAACAAAGTCAAGAATACGAGCAGCGTCACAGCACTAATCATCTGTATCAA  GATAATAGACCTTTTCCTTGAAATTGTCATTTCATCTNTCCCAGGAAGTCACGA  CCCGCGACTTGCCTACTACGAGTCCCCTGTGCTCTTGTTGATGTTACNGGATTC  CTAAAGTGATCAATAAACTGATTAATGCCCAAAAAAAAAAAAAAAAAAAAAAAA  AAAAAAAAAAAAAAAAAA |
| --- | --- |

**Figure S5** Number of spots labelled by anti-phosphoserine (**a-d**) and anti-*O*-GlcNAc (**e-g**) antibodies

**
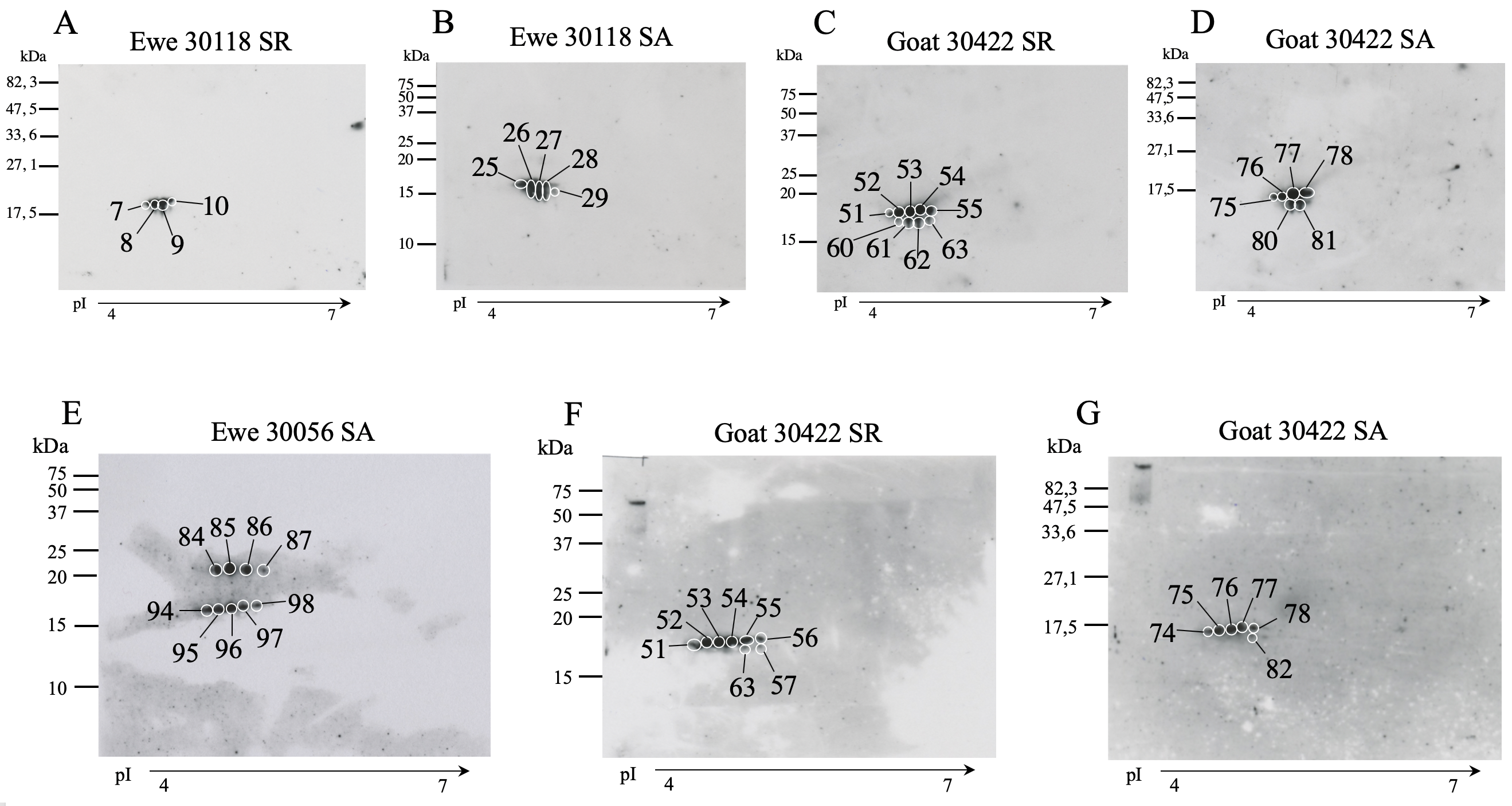
**

**Figure S6** Control of Q5 and CTD110.6 antibodies specificity. **a** western-blot with anti-phosphoserine Q5 antibody (Qiagen) with (+) or without (-) alkaline phosphatase treatment. **b** competition assay between CTD110.6 (1/5,000 dilution) and 1M GlcNAc after 2D electrophoresis of 30 μg of proteins - Coomassie blue staining of a similar gel, Ponceau red staining of the membrane, photographic film – control 5 ng of BSA-*O*-GlcNAcylated (Fisher Scientific).

**
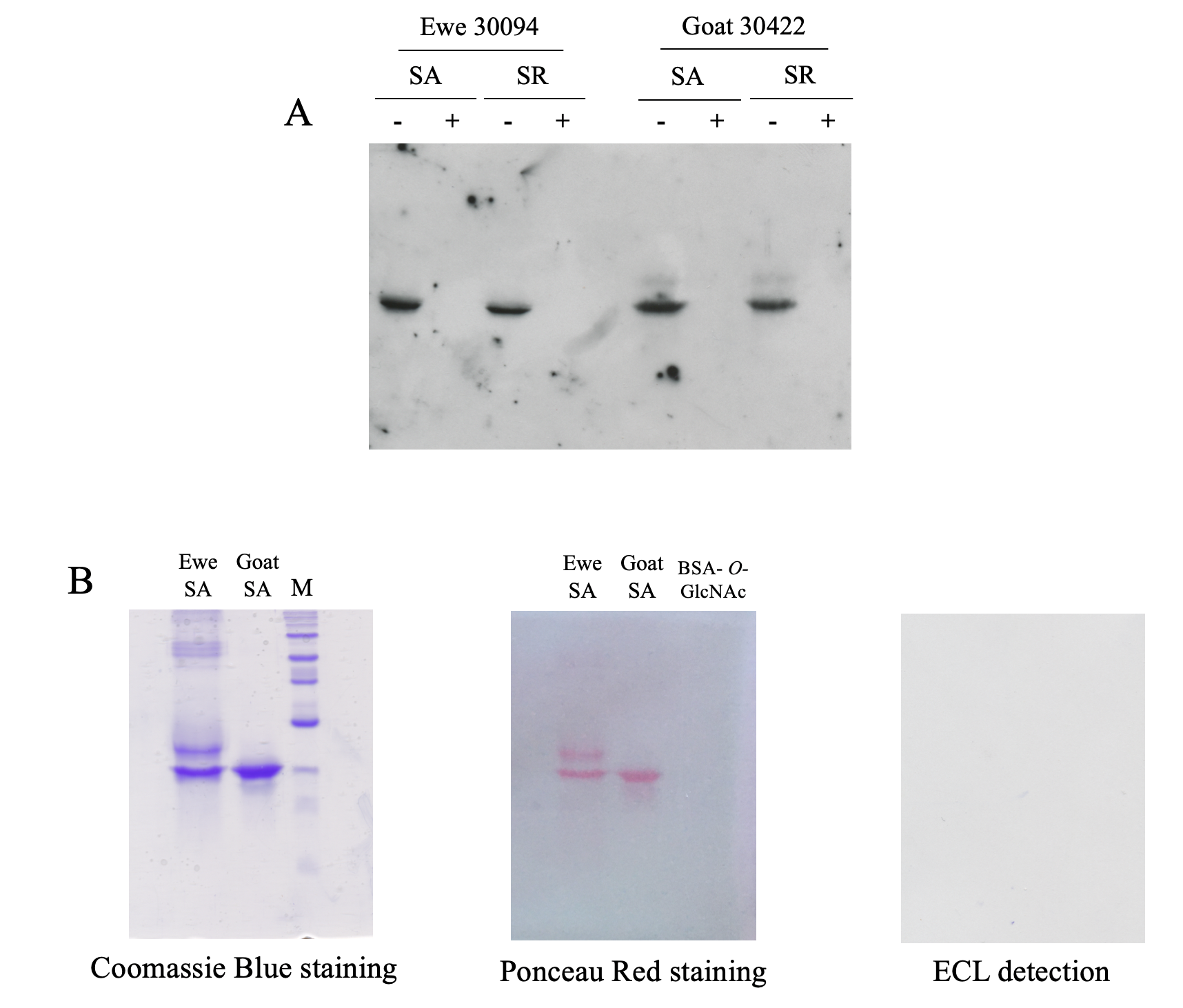
**

**Figure S7** Immunodetection of phosphothreonine proteins by western-blot with Q7 Antibody (Qiagen). Pig OBP (15 μg) was used as positive control (Nagnan-Le Meillour *et al.,* 2009).


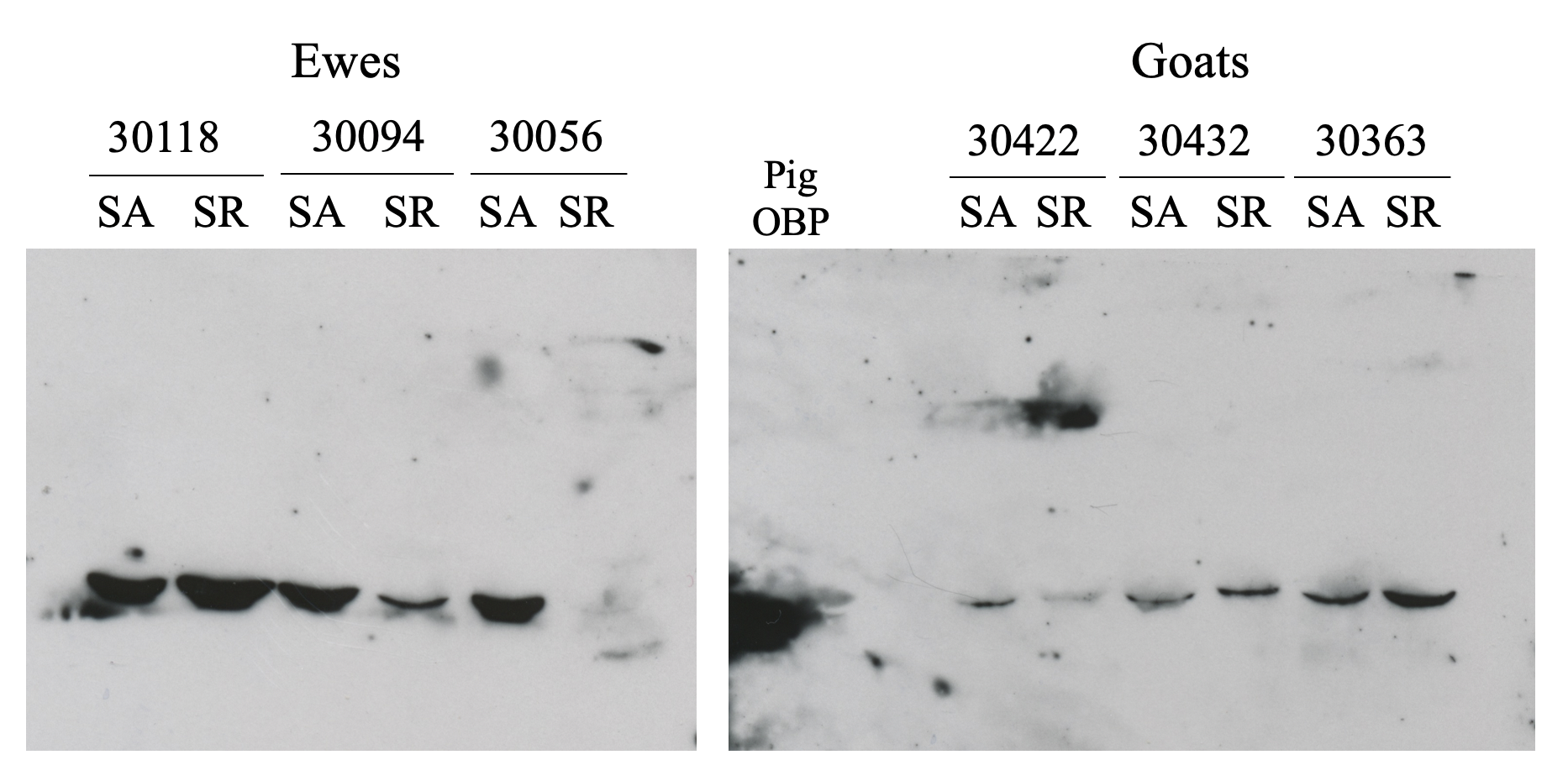


**Figure S8** Comparison between naked and phosphorylated MS/MS spectra of the same peptide (THYIASSNTEK**T**GENGPFNVYLR). **a** MS/MS spectrum of naked peptide 18-40 of Oari-OBP2 in spot 27 of ewe 30118 in SA (ion score 81.8 and identity score 42.2). **b** MS/MS spectrum of peptide 18-40 phosphorylated at Thr29 position of Oari-OBP2 (same spot, same ewe, same season, ion score 41.5 and identity score 42.4). Spectra were acquired with Scaffold viewer (Thermo Scientific®).


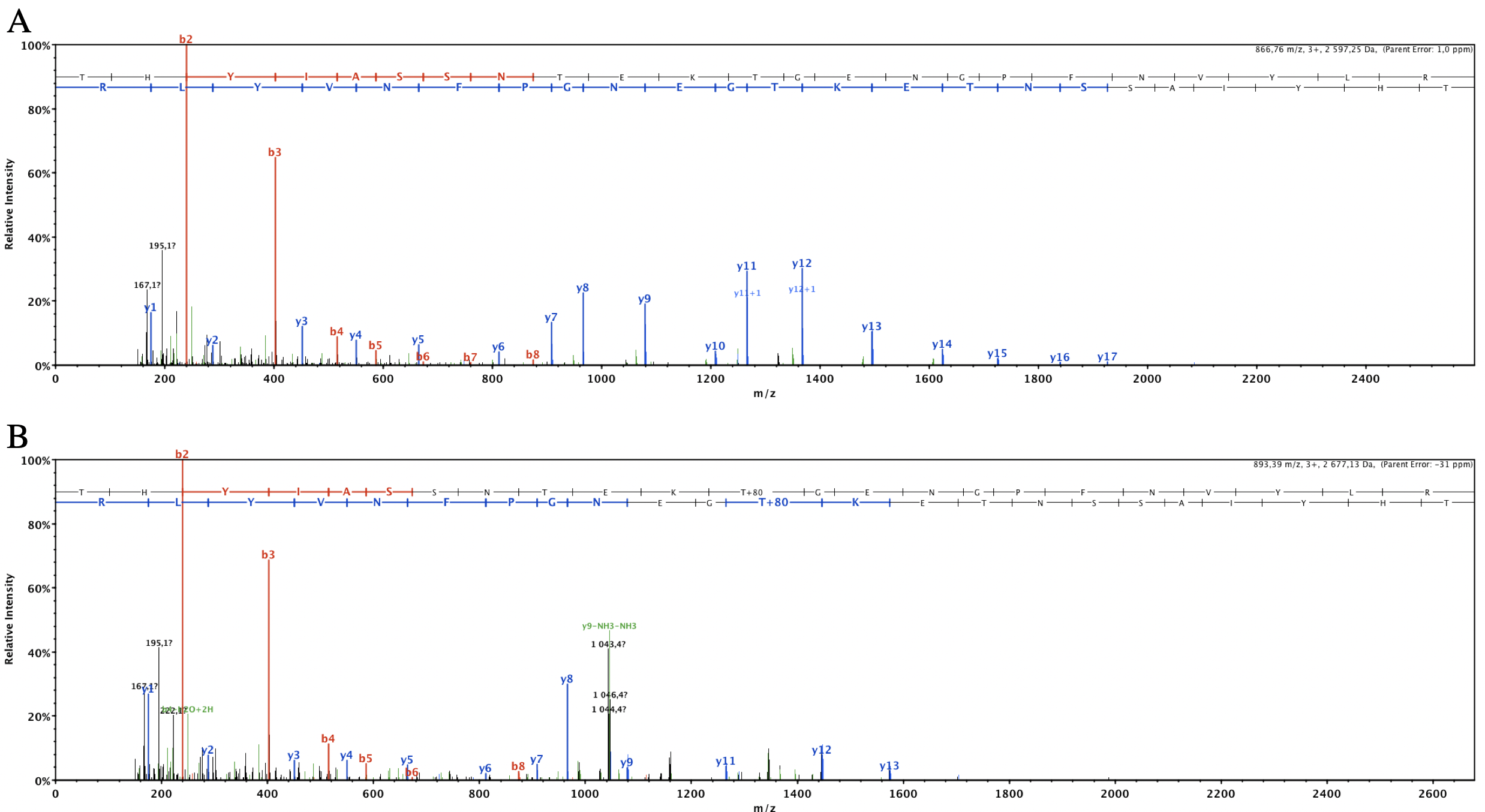

Supplement: Supplementary file 1 — Additional file 1: Table S1. Odorant-binding proteins identified in ewe 30,118 olfactory secretome in SR and SA by nano-LC-MS/MS. Table S2. Odorant-binding proteins identified in ewe 30,094 olfactory secretome in SR and SA by nano-LC-MS/MS. Table S3. Odorant-binding proteins identified in ewe 30,056 olfactory secretome in SR and SA by MALDI-TOF MS. Table S4. Odorant-binding proteins identified in goat 30,363 olfactory secretome in SR and SA by nano-LC-MS/MS or MALDI-TOF MS. Table S5. Odorant-binding proteins identified in goat 30,422 olfactory secretome in SR and SA by MALDI-TOF MS. Table S6. Odorant-binding proteins identified in goat 30,432 olfactory secretome in SR and SA by nano-LC-MS/MS. Table S7. Monitoring of progesterone concentration in blood of the ewes and goats used in this study. Table S8. Primers used for amplification of the major OBPs expressed in ewe and goat olfactory secretome. Figure S1. Sequence alignment of predicted lipocalins from sheep and goat genomes (BlastX searches). Figure S2. Two-dimensional electrophoresis of soluble proteins extracted from nasal mucus of ewes 30,094 and 30,056 in sexual rest (SR) and sexual activity (SA) periods. Figure S3. Two-dimensional electrophoresis of soluble proteins extracted from nasal mucus of goats 30,422 and 30,432 in sexual rest (SR) and sexual activity (SA). Figure S4. Full-length nucleotide and translated amino acid sequences of Chir-OBP2 and Chir-OBP4 obtained by RACE-PCR. Figure S5. Spot numbers labelled by anti-phosphoserine (a-d) and anti-O-GlcNAc (e-g) antibodies. Figure S6. Control of Q5 and CTD110.6 antibodies specificity. Figure S7. Immunodetection of phospho-threonine proteins by western-blot with Q7 Antibody (Qiagen). Figure S8. Comparison between naked and phosphorylated MS/MS spectra of the same peptide (THYIASSNTEKTGENGPFNVYLR). [file 12864_2019_6194_MOESM1_ESM.docx]
